# Solvent-dependent Sensitization and Photophysical Behavior of Ir^III^-Eu^III^ Bimetallic Complexes

**DOI:** 10.1007/s10895-026-04895-1

**Published:** 2026-08-11

**Authors:** Felipe S. M. Canisares, Vytor C. Oliveira, Alessandro B. S. Garcia, João Honorato, Javier A. Ellena, Marian R. Davolos, Ana M. Pires, Sergio A. M. Lima

**Affiliations:** 1 Institute of Chemistry, São Paulo State University (UNESP), Araraquara, SP Brazil; 2 Institute of Chemistry, University of São Paulo (USP), São Paulo, SP Brazil; 3 Institute of Physics, University of São Paulo (USP), São Carlos, SP Brazil; 4 Department of Chemistry and Biochemistry, School of Technology and Sciences, São Paulo State University (UNESP), R. Roberto Simonsen, 305, 19060-900, Presidente Prudente, SP Brazil; 5 Institute of Biosciences, Humanities and Exact Sciences, São Paulo State University (UNESP), São José Do Rio Preto, SP Brazil

**Keywords:** Iridium, Europium, Bimetallic complexes, Solvent effects, Energy transfer, Photoluminescence, Judd–Ofelt parameters

## Abstract

The photophysical properties of a heterobimetallic Ir^III^–Eu^III^ complex were investigated in solvents with different polarity, coordinating ability, and vibrational characteristics to elucidate the factors governing Eu^III^ sensitization. Steady-state and time-resolved luminescence measurements revealed that the solvent profoundly influences both the donor-to-acceptor energy-transfer process and the europium-centered emission. The apparent Ir^III^ → Eu^III^ energy-transfer rate constants (k_ET_) and donor-to-acceptor energy-transfer efficiencies (η_ET_) were estimated from the residual ^3^MLCT emission lifetimes (τ_q_) of the Ir^III^–Eu^III^ complex and the corresponding Ir^III^–Gd^III^ analog (τ_u_). The results showed a general tendency toward higher energy-transfer rates and efficiencies in solvents with high polarity, coordinating ability, and free of O–H oscillators, reaching k_ET_ values up to 2.6 × 10^7^ s^−1^ and η_ET_ values up to 90% in DMSO. Solvent polarity was found to indirectly modulate the energy transfer (ET) process by influencing the energies of the excited states and the donor–acceptor electronic coupling. In contrast, protic solvents strongly quenched Eu^III^ emission due to the presence of high-energy O–H oscillators, despite moderate ET efficiencies. Degassing experiments performed in dichloromethane and chloroform demonstrated that dissolved oxygen has only a minor effect on the η_ET_ efficiency. The results reveal a complex interplay between solvent polarity, coordinating ability, donor–acceptor gap, and non-radiative deactivation pathways, providing new insights into the design of efficient *d–f* heterobimetallic luminescent systems.

**Supplementary Information:**

The online version contains supplementary material available at https://doi.org/10.1007/s10895-026-04895-1.

## Introduction

Lanthanide(III) ions (Ln^III^) exhibit sharp 4*f*–4*f* electronic transitions, yielding naturally high-purity emission colors, yet their Laporte-forbidden character results in intrinsically weak absorption [1–3]. To overcome this limitation, Weissman [4] (1942) demonstrated that organic ligands can transfer absorbed energy to Ln^III^ centers—the so-called *antenna effect*. In this mechanism, the ligand absorbs UV–Vis light and transfers the excitation energy to the lanthanide, enabling its characteristic emission. Weissman also revealed that the sensitization efficiency depends on the ligand environment, solvent, and temperature. Since then, extensive efforts have been made to design improved sensitizers to enhance the luminescence of lanthanide complexes.

Over the past two decades, *d*-metal complexes have emerged as efficient sensitizers for lanthanide(III) ions owing to their high chemical and photostability and long-lived triplet excited states[5, 6]. Among them, Ir^III^-based complexes are in the spotlight, featuring broad absorption bands [7] that enable sensitization of diverse lanthanides, with emissions spanning from the near-infrared to the blue region. In these heterometallic systems, the Ir^III^ moiety governs the photophysical behavior by absorbing light and transferring energy from its triplet metal-to-ligand charge transfer (^3^MLCT) state to the Ln^III^ center. Beyond their attractive luminescent properties, Ir^III^–Ln^III^ assemblies integrate the magnetic and catalytic characteristics of lanthanides with the electroluminescent and photocatalytic attributes of iridium, making them attractive for multifunctional applications [8].

The first Ir^III^–Ln^III^ bimetallic complex was reported by De Cola et al. [9] in 2005 and featured a Eu^III^ center. In this study, partial energy transfer from the Ir^III^ complex to the Eu^III^ ion resulted in white-light emission. In 2009, Chen et al. [10] demonstrated energy transfer from [Ir(dfppy)_2_(bpm)Cl] to Nd^III^, Yb^III^, and Er^III^ ions using fluorescence titration. In this process, the luminescence of the Ir^III^ complex was quenched on the addition of lanthanide ions, accompanied by the appearance of characteristic near-infrared emission from the lanthanides. In 2011, Sykes and co-workers [11] provided the first evidence of Tb^III^ sensitization by a visible-emitting Ir^III^ complex, whose excited-state energy exceeded 22000 cm^−1^. Shortly thereafter, in 2012, Yang’s group [12] reported an Ir^III^–Gd^III^ bimetallic complex designed for dual-mode phosphorescence and magnetic resonance (MR) imaging, exhibiting intense sub-microsecond phosphorescence in aqueous media. Although energy transfer to Gd^III^ is not feasible due to its high-lying excited state (~ 32000 cm^−1^), the complex showed low cytotoxicity, effective cellular uptake, and specific mitochondrial staining, underscoring its potential as a metal-based molecular probe.

In 2014, Zheng et al*.* [13] synthesized an Ir^III^–Dy^III^ complex supported by phosphonate ligands, which exhibited both photoluminescence and field-induced slow magnetic relaxation (single-molecule magnet behavior) arising from the Ir^III^ and Dy^III^ centers, respectively. This work highlighted the potential of Ir^III^–Ln^III^ assemblies as bifunctional magneto-optical materials. Later, in 2016, Ward et al*.* [14] demonstrated the application of Ir^III^–Eu^III^ complexes as ratiometric chemical probes, in which only the Eu^III^-centered emission was selectively quenched upon interaction with 2-diisopropylaminoethyl ethyl methylphosphonate (VO). More recently, in 2020, Su et al*.* [15] reported a trimetallic Ir^III^–Eu^III^–Yb^III^ complex that exhibited efficient near-infrared down-conversion (NIR-DCL) and visible up-conversion luminescence (UCL) under ambient conditions. In 2026, we demonstrated the use of an Ir^III^-Eu^III^ bimetallic complex grafted onto silica particles for ratiometric oxygen sensing and cell imaging [16]. The decorated particles exhibited low cytotoxicity and, by confocal microscopy, were shown to be internalized by the Huh-7.5 cells, which maintained their luminescent properties, with green and red emissions detected separately using the same excitation source.

This timeline underscores the versatility of bimetallic and multimetallic Ir^III^–Ln^III^ complexes for diverse applications, attributable to the distinct physicochemical properties of each metal center. Significant research efforts have focused on elucidating the energy-transfer (ET) mechanisms between Ir^III^ chromophores and Ln^III^ ions, correlating ET efficiency with both the chemical structure of the cyclometalating ligands coordinated to Ir^III^ and the nature of the bridging ligand connecting the two metal centers.

The first full sensitization of Eu^III^ emission by an Ir^III^-based complex was described by Chen et al*.* [17] in 2008, through the synthesis of two bimetallic complexes: {[(dfppy)_2_Ir(μ-phen5f)]_3_EuCl}Cl_2_ and [(dfppy)_2_Ir(μ-phen5f)Eu(TFAcA)_3_], where dfppy is 2-(4′,6′-difluorophenyl)pyridinato-N,C^2^’, phen5f is 4,4,5,5,5-pentafluoro-1-(1′,10′-phenanthrolin-2′-yl)pentane-1,3-dionate, and TFAcA is trifluoroacetylacetonate. Photophysical studies indicated that the intense red emission from Eu^III^ was mediated by a triplet metal-to-ligand charge transfer (^3^MLCT) state of the Ir^III^ complex. However, in the absence of single-crystal data, the energy-transfer mechanism remained unclear.

Subsequently, Tart et al*.* [18] investigated a series of Ir^III^–Eu^III^ complexes incorporating different bridging ligands and observed partial energy transfer, as both the Ir^III^-based moiety phosphorescence and the characteristic narrow Eu^III^ emission bands were simultaneously detected, resulting in an overall orange emission. This study emphasized the pivotal role of the bridging ligand in Eu^III^ sensitization. The crystal structure of a representative complex enabled the determination of the Ir···Eu distance, which, together with photophysical and computational analyses, suggested that the ET process is governed by an exchange mechanism facilitated by π-stacking interactions between the components. In addition, the authors highlighted that conformational flexibility in solutions may significantly influence the sensitization efficiency.

Sykes et al. [19] investigated a series of Ir^III^–Gd^III^ complexes. They observed partial luminescence quenching on substitution of Gd^III^ with Eu^III^, which was attributed to the formation of a charge-separated state. This observation suggested that an electron-transfer–based ET pathway may contribute to Eu^III^ sensitization. In a subsequent study, Sykes et al. [20] examined Ir^III^–Eu^III^ and Ir^III^–Tb^III^ complexes, where time-resolved luminescence measurements revealed the formation of a photoinduced charge-separated species, {Ir^4+^}*–(NN ligand)•⁻, upon excitation. These findings support a photoinduced electron transfer (PET) mechanism as the dominant energy-transfer pathway in these systems, operating concurrently with the conventional exchange-mediated process.

As discussed above, the energy of the excited state of the Ir^III^ complex (donor, D) plays a crucial role in sensitizing the emissive states of Ln^III^ ions. Recently, we demonstrated that the minimum donor-state energy required for efficient sensitization of Eu^III^ by an Ir^III^-based ligand complex is 19103 cm^−1^ in a rigid environment, such as that of PMMA films.^19^ However, the efficiency of the energy transfer process in solution remains unclear. It is strongly influenced by solvent properties, as demonstrated by Tart et al.^16^ and previously suggested by Weissman in 1942.^3^ Motivated by these findings, we report herein two bimetallic Ir^III^–Ln^III^ complexes (Ln^III^ = Gd^III^, Eu^III^) and investigate the influence of solvent polarity, coordinating ability, and vibrational characteristics on their photophysical behavior. By correlating the energy gap between the donor (^3^MLCT) and acceptor (^5^D_0_ or ^5^D_1_) states with solvent-dependent variations in energy-transfer efficiency, this study provides new insights into the interplay between molecular structure, solvent effects, and energy-transfer mechanisms. This work aims to deepen the understanding of sensitization pathways in Ir^III^–Ln^III^ systems and contribute to the design of more efficient luminescent probes and multifunctional materials.

## Experimental Section

### **Materials and Reagents**

Methanol (CH_3_OH, Synth, 99.5%), iridium(III) chloride hydrate (IrCl_3_ · xH_2_O, Aldrich, 99.99%), 2-ethoxyethanol (C_4_H_10_O_2_, Aldrich, 99.0%), dichloromethane (CH_2_Cl_2_, Synth, 99.99%), 2-(2,4-difluorophenyl)pyridine (dfppy—C_11_H_7_F_2_N, Aldrich, 97%), and 2,2’-bipyridine-3,3’-dicarboxylic acid (bpdc—C_12_H_8_N_2_O_4_, Aldrich, 97%). The Ir^III^ complex [Ir(fppy)_2_(bpdc)] (Ir^III^-p) and the Ir^III^-Eu^III^ bimetallic complex [{Ir(dfppy)_2_(µ-bpdc)}_3_Eu_2_]Cl_3_·nH_2_O·mCH_3_OH were synthesized and characterized in a previous study [21].

### **Instrumentation**

FTIR spectra were recorded using an IRAffinity-1 Shimadzu spectrometer over the 4000–400 cm^−1^ range, with 100 scans and a resolution of 1 cm^−1^, in ATR mode. Elemental analyses (CHN) were performed on a PerkinElmer 2400 Series II. Luminescent measurements were performed using a Horiba Jobin Yvon Fluorolog-3 Spectrofluorometer, model Fluorolog-3–221, equipped with a continuous 450 W Xenon lamp, double-excitation and double-emission monochromators, and an R 928 Hamamatsu photomultiplier. All spectra were corrected using detector-mode correction. The ^3^MLCT emission lifetime measurements were performed using time-correlated single-photon counting (TCSPC) with an IBH Fluorocube system interfaced to a Horiba FluoroHub + controller, with excitation at 339 nm. The absolute emission quantum yields were measured using an integrating sphere with an absolute error of 10% at 320 nm excitation for all tested solutions. Single-crystal X-ray diffraction data were collected at 100 K on a Rigaku Synergy-S diffractometer equipped with a Hybrid Photon Counting (HPC) detector, using Cu Kα radiation (λ = 1.54184 Å) from a microfocus sealed X-ray tube. A suitable single crystal was selected and cooled under a stream of nitrogen gas using an Oxford Cryosystems cooling device. Data collection, indexing, integration, and scaling were performed using the CrysAlisPro software package. The structure was solved by intrinsic phasing and refined by full-matrix least-squares methods on F2 using SHELXT and SHELXL [22], respectively, within the Olex2 interface [23].

### **Synthesis of Bimetallic Ir**^**III**^**-Ln**^**III**^** Complexes:**

A mass of the iridium complex, Ir^III^-p, was dissolved in 15 mL of methanol, after which the corresponding LnCl_3_ (Ln^III^ = Gd^III^ and Eu^III^), previously dissolved in methanol, was added to the iridium complex solution in a 1.5:1 Ir^III^: Ln^III^ molar ratio. The mixture was stirred for 4 h at room temperature. Subsequently, the solution was heated in an open system to reduce its volume, and the complex was precipitated by adding water. The precipitate was then collected by centrifugation, washed several times with water, and dried in a desiccator.

### **[{Ir(dfppy)**_**2**_**(µ-bpdc)}**_**3**_**Gd**_**2**_**]Cl**_**3**_**·nH**_**2**_**O·mCH**_**3**_**OH:**

Yield: 77.8%. FTIR – ATR (ʋ_max_/cm^−1^): 1598 (_υ_COO^−^)_ass_, 1572, 1558 (_υ_C = N)_pyridine_, 1385 (_υ_COO^−^)_sym_. Elemental analysis; found(calcd.) C: 40.79%(40.17%), H: 2.71%(2.66%), N: 5.67%(5.41%) to C_104_H_82_Cl_3_Gd_2_F_12_Ir_3_N_12_O_24_ ‒ [{Ir(dfppy)_2_(µ-bpdc)}_3_Gd_2_]Cl_3_·10H_2_O·2CH_3_OH. Fluorescence: excitation λ_max_ (in aerated DCM solution, 1.0 × 10^–5^ mol L^−1^) 451 nm, emission 560 nm. Quantum yield (Φ_fl_) 40.5%, τ = 637.2 ns.

### **[{Ir(dfppy)**_**2**_**(µ-bpdc)}**_**3**_**Eu**_**2**_**]Cl**_**3**_**·nH**_**2**_**O·mCH**_**3**_**OH:**

Yield: 86.5%. FTIR – ATR (ʋ_max_/cm^−1^): 1598 (_υ_COO^−^)_ass_, 1572, 1558 (_υ_C = N)_pyridine_, 1384 (_υ_COO^−^)_sym_. Elemental analysis; found(calcd.) C: 39.45%(39.39%), H: 2.87%(2.86%), N: 5.90%(5.30%) to C_104_H_90_Cl_3_Eu_2_F_12_Ir_3_N_12_O_28_ ‒ [{Ir(dfppy)_2_(µ-bpdc)}_3_Eu_2_]Cl_3_·14H_2_O·2CH_3_OH. Fluorescence: excitation λ_max_ (in aerated DCM solution, 1.0 × 10^–5^ mol L^−1^) 451 nm, emission Ir^III^ component 560 nm, Eu^III^ component 616 nm. Quantum yield (Φ_fl_) 31.7%, τ_Ir_ = 176.5 ns, and τ_Eu_ = 0.82 ms.

## Results and Discussion

### Structural Characterization

**Fig. 1:**
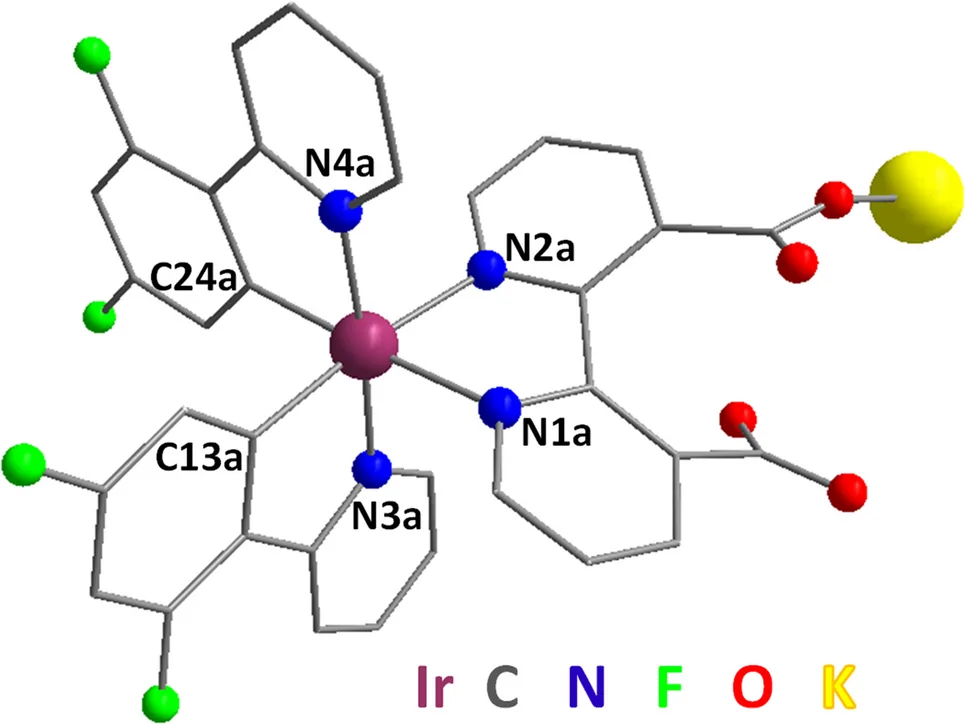
Crystal structure of Ir^III^-p. H atoms were removed for clarity

The Ir^III^ complex [Ir(dfppy)_2_(bpdc)] (Ir^III^–p) and the bimetallic Ir^III^–Eu^III^ complex [{Ir(dfppy)_2_(μ-bpdc)}_3_Eu_2_]Cl_3_·nH_2_O·mCH_3_OH were previously synthesized and characterized [19]. Crystals of Ir^III^-p suitable for X-ray diffraction were obtained by slow diffusion of hexane into a dichloromethane solution of the complex. Crystallographic data are summarized in Table S1 (Supporting Information), and atomic coordinates, bond lengths, and angles are provided in Tables S2–S8. The molecular structure is shown in Fig. 1. Ir^III^-p crystallizes as yellow triclinic crystals in the P-1 space group. The carboxylate groups are fully deprotonated, and K⁺ ions bridge adjacent Ir^III^-p units (Figure S1), forming one-dimensional chains. Two water molecules also coordinate each K^+^ ion. The complex contains C^N cyclometalating ligands in a trans N–Ir–N configuration, consistent with a room-temperature synthesis [24, 25]. The angle between the nitrogen atoms of the coordinated dfppy cyclometalating ligands is nearly linear, measuring 174.959(139)° (N3a_dfppy_-Ir1a-N4a_dfppy_). The complex adopts a pseudo-octahedral coordination geometry, with three chelating ligands arranged around the Ir^III^ center. The Ir–C bond lengths to the cyclometalating ligands, Ir-C13a_dfppy_ and Ir-C24a_dfppy_, are 2.0139(37) and 2.0059(36) Å, respectively. The Ir-N bond lengths to the ancillary bpdc ligand exhibit slightly less variation, measuring 2.1334(27) Å (Ir–N2a_bpdc_) and 2.1256(27) Å (Ir–N1a_bpdc_), which is consistent with strong coordination while maintaining geometric rigidity.

Following the elucidation of the molecular structure of Ir^III^-p, its coordination behavior toward Gd^III^ and Eu^III^ ions was investigated to afford the corresponding bimetallic complexes. Since no single crystals of the bimetallic species were obtained, a combination of complementary techniques was employed to confirm their formation. The stoichiometries of the newly prepared Ir^III^-Gd^III^ complex and the previously reported Ir^III^-Eu^III^ analogue^19^ were confirmed by elemental analysis, with experimental C, H, and N contents in good agreement with the calculated data (see "Experimental Section" section). The FTIR spectrum revealed that the Ir^III^ complex coordinates to the Gd^III^ center through the carboxylate oxygen atoms. Upon coordination, the antisymmetric stretching vibration of the carboxylate group, ν_asym_(COO^−^), shifted slightly from 1601 to 1598 cm^−1^, overlapping with the C = N stretching band. Meanwhile, the symmetric stretching vibration, ν_sym_(COO^−^), shifted to higher energy, from 1374 to 1385 cm^−1^. Other characteristic bands associated with C = N and C = C vibrations remained unchanged after coordination (Figure S2). Based on the FTIR data, the carboxylate groups of the Ir^III^-p moiety coordinate to the Gd^III^ ion in a monodentate mode, as indicated by the separation between ν_asym_(COO^−^) and ν_sym_(COO^−^) (Δ = 213 cm^−1^), which is larger than that observed for the free ionic ligand Na_2_bpdc (Δ = 205 cm^−1^; Figure S3) [26]. This behavior is consistent with that previously reported for the analogous Ir^III^-Eu^III^ complex [19].

To determine the actual stoichiometric ratio between Ir^III^ and Ln^III^ ions in the synthesized bimetallic complexes, luminescence titration experiments were performed.^17^ An ethanolic solution of the Ir^III^ complex (Ir^III^-p, 0.12 mM) was titrated with a 3.06 mM LnCl_3_ solution (Ln = Eu or Gd). An emission spectrum was recorded after each addition of a 100 μL aliquot of the lanthanide solution. Upon titration with EuCl_3_, both progressive luminescence quenching and a red shift of the Ir^III^-moiety emission band are observed, along with the appearance of narrow Eu^III^-centered emission bands in the red region, confirming energy transfer between the two metal centers (Figure S4A) under excitation of the Ir^III^ complex (λ_ex_ 320 nm). To better understand these spectral changes, the energy of the Ir^III^ emission maximum was plotted against the Ir^III^/Eu^III^ molar ratio (Figure S4B). Notable variations occurred up to a ratio of 1.48, beyond which no further spectral changes were observed. This behavior indicates that the stoichiometric ratio in the resulting Ir^III^-Eu^III^ complex is approximately 1.5:1, in agreement with the expected composition. In contrast, titration with GdCl_3_ increased the emission intensity of the Ir^III^ complex, a trend that will be explored in the next section. This enhancement was also accompanied by a red shift in its emission band (Figure S4C). Given that the first excited state of Gd^III^ lies at approximately 32000 cm^−1^, energy transfer from the Ir^III^ luminophore to Gd^III^ is energetically unfavorable. As a result, only a broad, structureless emission band is observed following GdCl_3_ addition. Spectral changes are detected up to an Ir^III^/Gd^III^ molar ratio of 1.73 (Figure S4D), consistent with the expected stoichiometry and with the behavior observed in the Ir^III^-Eu^III^ system.

### Photoluminescence Study

**Fig. 2:**
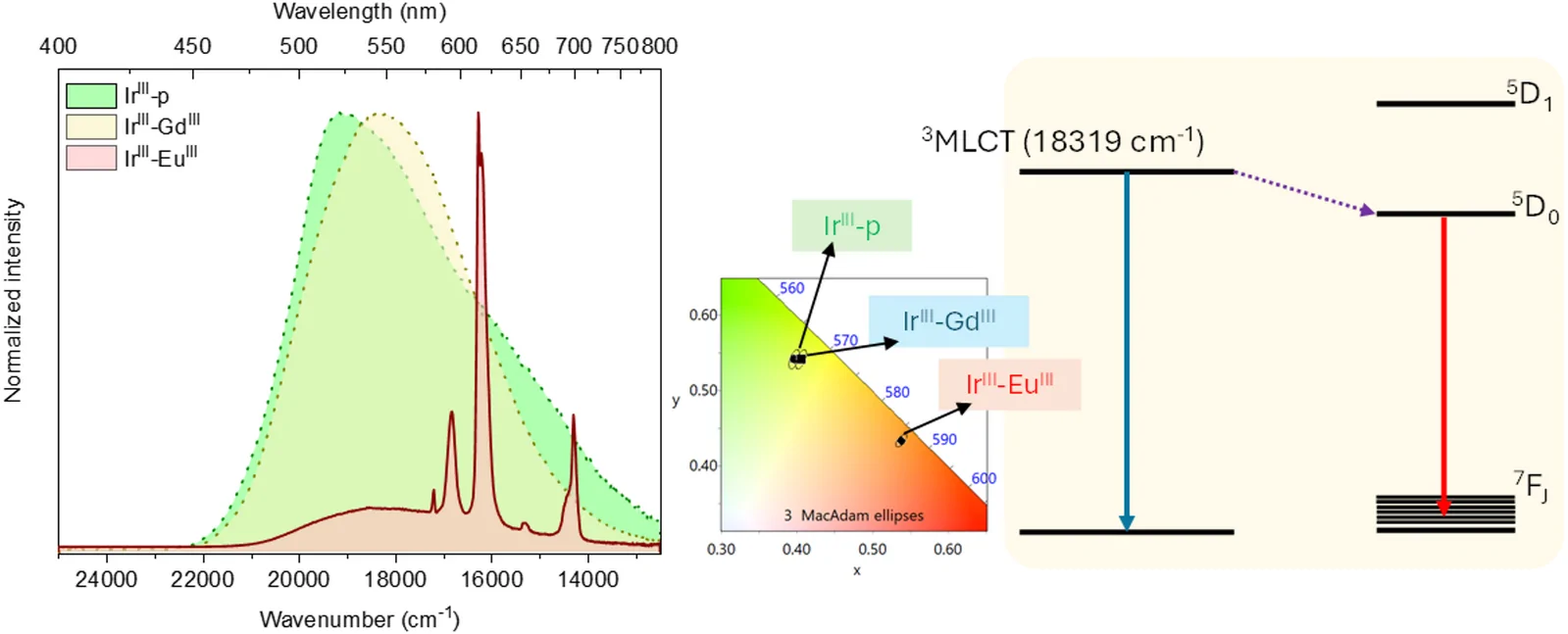
Emission spectra of Ir^III^-p, Ir^III^-Gd^III^, and Ir^III^-Eu^III^ complexes acquired in the solid state. All measurements were carried out with a bandpass of 2.5 nm for both Ex and Em, with a 0.5 nm increment, an integration time of 0.5 s, and excitation at 326 nm. The color diagram represents the emission of the three complexes. The energy levels of the donor state (^3^MLCT) and selected states of the Eu^III^ ion are also represented

The emission behavior of the complexes was initially evaluated in the solid state (Fig. 2). The monometallic Ir^III^ complex (Ir^III^–p) displays a broad, featureless emission band spanning 450–800 nm, effectively covering the entire visible region. The bimetallic Ir^III^–Gd^III^ complex exhibits a similar unstructured emission profile over the same spectral range, but with a notable redshift in the emission maximum: from 522 nm in Ir^III^–p to 545 nm in the Ir^III^–Gd^III^ complex (Fig. 2). Such red shifts in *d-f* heterobimetallic systems have been previously reported and are commonly attributed to enhanced spin–orbit coupling caused by the presence of a second metal center [10, 27]. Despite this shift, the change in dominant emission wavelength is modest and visually imperceptible, as confirmed by the corresponding CIE color diagram (Fig. 2).

The emission spectrum of the Ir^III^-Eu^III^ complex revealed partial energy transfer from the Ir^III^ luminophore to the Eu^III^ ion, as evidenced by the coexistence of a broad Ir^III^-based band centered at 545 nm and sharp Eu^III^ emission bands (Fig. 2). According to the empirical Latva criterion[28], efficient energy transfer requires the donor excited state to be at least ~ 2500 cm^−1^ above the acceptor emitting level. In our previous work^19^, we estimated the minimum energy of the donor for full sensitization in heterometallic Ir^III^-Eu^III^ complexes to be 19103 cm^−1^, assuming a rigid system with no solvent effects. In the present system, the ^3^MLCT state of the Ir^III^-p chromophore, estimated from the solid-state emission spectrum of the Ir^III^-Gd^III^ analog, is located at 18319 cm^−1^, corresponding to a gap of only 1092 cm^−1^ above the Eu^III 5^D_0_ emitting level. This small energy gap accounts for the incomplete observed energy transfer to ^5^D_0_. Other possible acceptor levels were not considered because the donor state (^3^MLCT) lies below them, such as ^5^D_1_ and ^5^D_2_, making energy transfer to them negligible. Nevertheless, the characteristic Eu^III^ emissions, arising from the ^5^D_0_ → ^7^F_J_ (J = 0–4) transitions, are clearly detected, and the combined presence of the broad Ir^III^ band and the narrow Eu^III^ bands results in an overall orange emission.

**Fig. 3:**
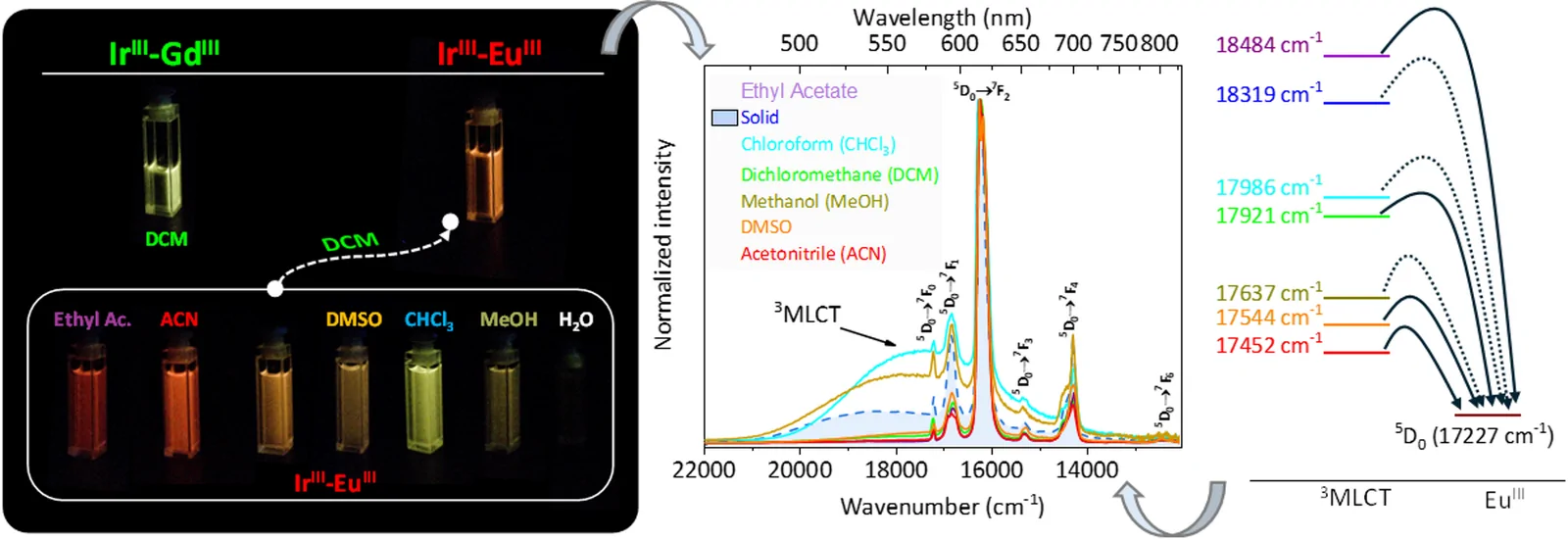
Images of the Ir^III^-Eu^III^ bimetallic complex in each tested solvent, and of the Ir^III^-Gd^III^ bimetallic complex in DCM under UV irradiation at λ_max_ ~ 350 nm; emission spectra of the Ir^III^-Eu^III^ complex and an energy diagram showing the triplet metal-to-ligand charge transfer (^3^MLCT) state, determined using the Ir^III^-Gd^III^ complex measured in different solvents (ACN: red; DMSO: orange; methanol: dark yellow; chloroform: light blue; powder sample: blue; DCM: green; and ethyl acetate: purple). The solution concentration was 1.0 × 10^–5^ mol·L^−1^. All measurements were carried out with a 2.5 nm bandpass for both Ex and Em, a 0.5 nm increment, and an integration time of 0.5 s. λ_ex_ 316 nm and λ_em_ 617 nm. Full arrows represent favorable energy transfer, and dashed arrows represent partial energy transfer. The ^3^MLCT energies were determined using the barycenter of the ^3^MLCT emission band

To gain deeper insight into the Eu^III^ sensitization mechanism in *d-f* bimetallic complexes, photoluminescence studies were conducted in various solvents to evaluate how the physicochemical properties of the solvents influence energy-transfer efficiency. Upon changing the solvent, distinct variations in emission color are observed, both visually and spectroscopically, as illustrated in Fig. 3. The corresponding excitation and emission spectra for the Ir^III^-p, Ir^III^-Gd^III^, and Ir^III^-Eu^III^ complexes in all investigated solvents are presented in Figure S5. The excitation spectra were also acquired by monitoring the ^5^D_0_ → ^7^F_2_ transition to ensure that sensitization occurs exclusively via the Ir^III^ chromophore, Figure S6. The solvent dependence of the emission of Ir^III^-p and Ir^III^-Gd^III^ was analyzed using the Lippert–Mataga approach, and no linear correlation was observed (Figure S7 and S8) [29]. Because a linear Lippert–Mataga relationship is expected when the emission properties depend primarily on solvent polarity, this result indicates that, in addition to general solute–solvent interactions, specific interactions with certain classes of solvents must also be considered [30], which in turn must influence the energy transfer to the Eu^III^ ion.

Another important insight can be obtained from Figure S4 by comparing the intensity changes observed for both bimetallic complexes relative to Ir^III^-p. Upon addition of Gd^III^ (Figure S4C), the emission intensity increases, as additional coordination of the carboxylate groups enhances molecular rigidity [31], thereby suppressing vibrational deactivation pathways. In contrast, the incorporation of Eu^III^ (Figure S4A) introduces an additional nonradiative deactivation pathway for the ^3^MLCT-emitting state, namely, energy transfer to the ^5^D_0_ excited level of Eu^III^. This pathway is absent in the Ir^III^–Gd^III^ complex because the excited states of Gd^III^ lie at much higher energies. Although Eu^III^ coordination also increases molecular rigidity, this effect is outweighed by energy transfer, resulting in overall quenching of the Ir^III^-moiety emission concomitant with the emergence of Eu^III^ emission. These observations provide further evidence for sensitization in this system. Spectral analyses reveal that the energy-transfer process is influenced not only by the energetic alignment between the donor and acceptor states of the bimetallic complexes but also by solvent-dependent factors, including stabilization of excited states, suppression or enhancement of nonradiative decay pathways, and potential coordination interactions with solvent molecules. Then, the solvent properties play a crucial role in modulating the efficiency of Eu^III^ sensitization. Furthermore, parameters such as dissolved oxygen content [32], which may quench excited states, and viscosity [33], which can modulate molecular motion and energy relaxation processes, may also contribute to the overall photophysical behavior of these bimetallic systems.

Although the donor-state energy could be directly determined from the Ir^III^-Eu^III^ complex in certain solvents, the Ir^III^-Gd^III^ complex was used as a reference to estimate the energy of the ^3^MLCT state responsible for Eu^III^ sensitization. This approach avoids interference from the Eu^III^-centered emission. It ensures that the measured emission originates exclusively from the Ir^III^-based luminophore upon coordination to a lanthanide ion. The resulting donor energy levels across different solvents are summarized in the energy diagram in Fig. 3 and Table 1.

**Table 1:** Maximum emission wavelength (λ_max_) and energy of the ^3^MLCT state of the Ir^III^-based complex in different solvents and in the solid state mesuared from the Ir^III^-Gd^III^ complex, energy gap (ΔE) between the ^3^MLCT state of the Ir^III^-based complex and the ^5^D_0_ level of the Eu^III^ ion, solvent dipole momentum (μ), and emission components observed in the emission spectra for each tested solvent and in the solid state

	λ_max_ (nm)	Energy (cm^−1^)	ΔE [^3^MLCT—^5^D_0_] (cm^−1^)	μ (D)	Emission spectra characteristics
*Water*	579	17271	44	1.85	^3^MLCT emission
*Acetonitrile (ACN)*	573	17452	225	3.92	Eu^III^ and residual ^3^MLCT
*DMSO*	570	17544	317	3.96	Eu^III^ and residual ^3^MLCT
*Methanol*	567	17637	410	1.70	Eu^III^ and ^3^MLCT emissions
*Dichloromethane (DCM)*	558	17921	694	1.60	Eu^III^ and residual ^3^MLCT
*Chloroform*	556	17986	759	1.04	Eu^III^ and ^3^MLCT emissions
*Powder sample*	545	18319	1092	-	Eu^III^ and ^3^MLCT emissions
*Ethyl Ac*	541	18484	1257	1.78	Eu^III^ emission

*The energy of the ^5^D_0_ level was assumed as 17227 cm^−1 41^

In ethyl acetate, energy transfer to Eu^III^ was efficient (Fig. 3), as virtually no emission from the Ir^III^ moiety was detected. The ^3^MLCT emission of the Ir^III^-Gd^III^ complex in this solvent is blue-shifted relative to the solid-state spectrum, occurring at 18484 cm^−1^ (541 nm) (Figure S5B). While this blue shift increases the energy gap between the donor and acceptor levels, it remains below the ~ 2500 cm^−1^ threshold set by Latva’s rule for highly efficient sensitization. Thus, the observed enhancement of Eu^III^ emission suggests that additional solvent-dependent effects—such as reduced nonradiative decay or altered coordination dynamics—also contribute to the improved energy-transfer efficiency in ethyl acetate.

In acetonitrile, the emission profile of the Ir^III^-Eu^III^ complex indicates highly efficient energy transfer, with the Ir^III^-based emission being virtually suppressed. In this solvent, the ^3^MLCT state lies solely at 17452 cm^−1^ (573 nm), only ~ 225 cm^−1^ above the Eu^III 5^D_0_ level (Fig. 3 and Table 1). Conversely, in chloroform, the ^3^MLCT energy is 17986 cm^−1^ (556 nm), 759 cm^−1^ above the Eu^III^ emissive state (Fig. 3 and Table 1). Despite this larger energy gap, energy transfer was markedly inefficient, and significant residual emission from the Ir^III^-centered luminophore was observed.

These results clearly demonstrate that the energy-transfer mechanism in solution for *d-f* bimetallic complexes cannot be rationalized solely based on the donor–acceptor energy gap, Table 1. The differences between the previous study^19^ in a rigid matrix and the present work arise from the distinct experimental conditions. In a rigid system, efficiency is primarily governed by the ^3^MLCT energy due to restricted conformational freedom and limited environmental effects. In solution, enhanced conformational flexibility and dynamic solvent interactions significantly influence energy transfer, with solvent properties such as polarity, coordination ability, and O–H oscillators playing key roles. Thus, studies in solutions provide a more realistic and comprehensive understanding of sensitization behavior under conditions relevant to practical applications. To more accurately assess the influence of solvent properties, the solvents were grouped according to their polarity and proton-donating ability: (i) aprotic polar solvents (ethyl acetate, acetonitrile, and DMSO), (ii) polar protic solvents (methanol and water), and (iii) solvents with dipole momentum lower than 1.7 D (chloroform and dichloromethane). The photophysical behavior of the complexes in these groups is discussed in detail below.

#### Aprotic Polar Solvents (Polarity > 1.7 D)

**Fig. 4:**
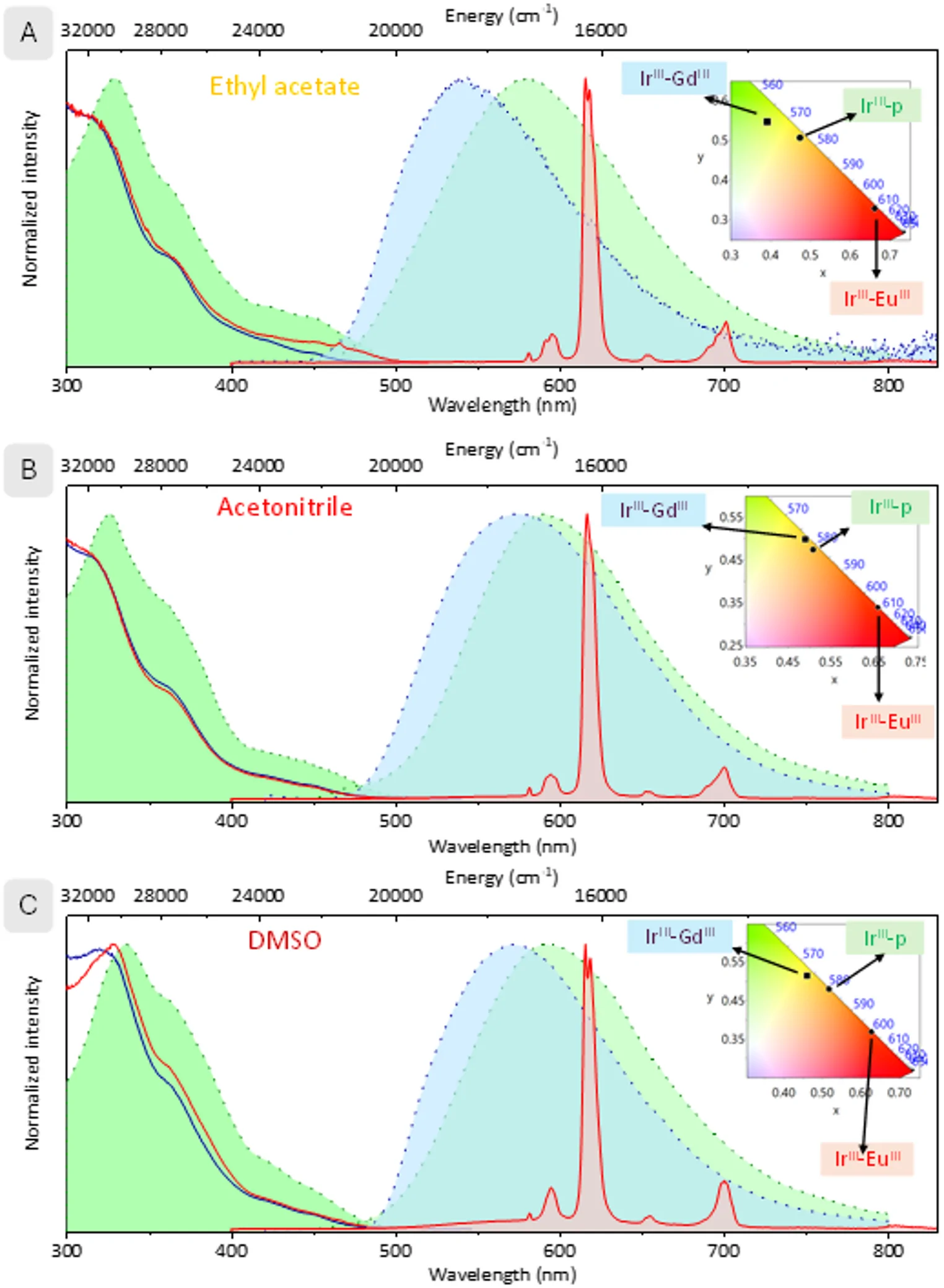
Excitation and emission spectra of Ir^III^-p (green), Ir^III^-Gd^III^ (blue), and Ir^III^-Eu^III^ (red) complexes measured in **A**) ethyl acetate, **B**) acetonitrile, and **C**) DMSO. Concentration in solution of 1.0 × 10^–5^ mol·L^−1^. All measurements were carried out with a bandpass of 2.5 nm for both Ex and Em, with a 0.5 nm increment and a 0.5 s integration time, using excitation at 316 nm

In aprotic polar solvents such as ethyl acetate, acetonitrile, and DMSO, the excitation spectra of the bimetallic exhibited similar features, with broad excitation bands from 300 to ~ 500 nm, Fig. 4. The high-energy bands, assigned to ^1^MLCT and ^1^LC transitions [34], are blue-shifted relative to the mononuclear Ir^III^-p complex, whereas the lower-energy ^3^MLCT absorptions also exhibit lower intensity. Notably, unlike in the solid state, the emission maximum of the Ir^III^-Gd^III^ complex was solvent-dependent and displayed a progressive blue shift compared to Ir^III^-p. This shift was particularly pronounced in ethyl acetate, the least polar solvent in this group (Fig. 4A).

Comparison of the emission spectra of the Ir^III^-Gd^III^ complex in solution with those in the solid state (Figure S5) reveals that, in less polar solvents, the emission profile closely matches that observed in the powder, indicating negligible solvent influence on the excited states of the Ir^III^ moiety. In contrast, a progressive redshift is observed in more polar solvents, likely due to stronger solvent-excited-state interactions [35]. This increased stabilization of the Ir^III^–centered moiety's excited state in polar environments may influence the energy alignment between the donor and acceptor levels, thereby affecting the efficiency of Eu^III^ sensitization in the corresponding bimetallic complex.

Considering the ratio of Ir^III^ and Eu^III^ emission intensities, ethyl acetate showed efficient energy transfer to the Eu^III^ ion, since the band associated with the Ir^III^ moiety is neglected. In this medium, the ^3^MLCT state of the Ir^III^-Gd^III^ complex is located at 1257 cm^−1^ above the Eu^III^ emissive level, the highest among the solvents tested, suggesting that the energy gap may be the primary factor governing sensitization efficiency (Fig. 3). However, emission spectra (Fig. 4) reveal that effective energy transfer occurs in all three aprotic polar solvents investigated, regardless of polarity. This trend is consistent with previous studies demonstrating that polar solvents can enhance energy transfer in Ln^III^-based systems [36]. Thus, in more polar solvents, the reduced energy difference between donor and acceptor is offset by solvent stabilization of the excited state, maintaining efficient energy transfer to Eu^III^.

The photophysical parameters of the complexes further evidence the influence of the solvent on the energy transfer process. Luminescent quantum yields (Φ) and emission lifetimes (τ) were determined at room temperature (Table 2), revealing that the Ir^III^-Gd^III^ bimetallic complex displays higher Φ and τ compared to the monocentered Ir^III^-p complex. This enhancement is attributed to increased structural rigidity upon coordination with the lanthanide ion in the bimetallic system, which likely suppresses vibrational relaxation and nonradiative deactivation pathways.^7^

**Table 2:** Photophysical properties of Ir^III^-p, Ir^III^-Gd^III^, and Ir^III^-Eu^III^ complexes measured in ethyl acetate, acetonitrile (ACN), dimethylsulphoxide (DMSO), methanol, water, dichloromethane (DCM), and chloroform. *Φ* is the overall emission quantum yield, ${\tau}_{Ir}$ is the emission lifetime measured from the Ir^III^ moiety emission, ${k}_{r}$ is the radiative decay rate, ${k}_{nr}$ is the nonradiative decay rate, ${\Phi}_{Eu}^{Eu}$ is the intrinsic emission quantum yield, ${\tau}_{Eu}$ is the lifetime of the Eu^III^ emission, and A_r_ and A_nr_ are the radiative and nonradiative decay rates of the ^5^D_0_ emissive state of the Eu^III^ ion. Concentration in solution of 1.0 × 10^–5^ mol·L^−1^

	Ir^III^-p				Ir^III^-Gd^III^				Ir^III^-Eu^III^					
	*Φ*[%]	*τ*[ns]	${k}_{r}$ [10^6^ s^−1^]	${k}_{nr}$ [10^6^ s^−1^]	*Φ*[%]	*τ*[ns]	${k}_{r}$ [10^6^ s^−1^]	${k}_{nr}$ [10^6^ s^−1^]	*Φ*[%]	${\Phi}_{Eu}^{Eu}$[%]	${\tau}_{Ir}$[ns]	${\tau}_{Eu}$[ms]	${A}_{r}$[s^−1^]	${A}_{nr}$[s^−1^]
*Aprotic polar solvents (polarity* > *1.7 D)*														
*Ethyl Ac*	2	11.9	1.7	82	7	265.2	0.3	3.5	25	37	47.5	0.81	457	777
*ACN*	2	179.6	0.1	5.4	30	557.8	0.5	1.3	47	53	163.2	1.18	453	394
*DMSO*	12	13.2	9.1	66	11	368.0	0.6	1.3	46	57	35.3	1.31	435	328
*Protic polar solvents (polarity* > *1.7 D)*														
*Methanol*	6	279.0	0.2	3.4	32	505.3	0.6	1.3	12	17	232.6	0.48	358	1725
*Water*	3	139.2	0.2	7.0	5	240.3	0.2	3.9	5	-	103.5	-	-	-
*Less polar solvents (polarity* < *1.7 D)*														
*DCM*	4	307.7	0.1	3.1	40	637.2	0.6	0.9	32	36	176.5	0.82	434	785
*Chloroform*	2	583.6	0.03	1.7	44	760.7	0.6	0.7	19	20	431.1	0.35	575	2281

* Radiative (k_n_) and nonradiative decay rates (k_nr_) of the Ir^III^-moiety emission in both Ir^III^-p and Ir^III^-Gd^III^ complexes were calculated from Φ and **τ**, using the following equations: Φ = k_r_/(k_r_ + k_nr_) and τ = 1/(k_r_ + k_nr_) [38]. Typically, the error of *Φ* measurements is considered to be 10%

A comparison of the photophysical properties of the Ir^III^-Gd^III^ and Ir^III^-Eu^III^ complexes reveals an increase in the emission quantum yield (Φ) and a decrease in the ^3^MLCT emission lifetime (τ) when Eu^III^ replaces Gd^III^. In the Ir^III^-Gd^III^ complex, emission originates solely from the ^3^MLCT state of the Ir^III^ moiety luminophore, as energy transfer to Gd^III^ is not feasible due to its high-lying excited states. Conversely, the emission of the Ir^III^-Eu^III^ complex arises predominantly from the Eu^III^ ion, following efficient sensitization by the Ir^III^ unit. Importantly, the triplet states associated with Ir^III^-based emission, as observed in Ir^III^-p and Ir^III^-Gd^III^, are highly susceptible to quenching by dissolved oxygen [37], leading to lower Φ. The decrease in the τ values after Eu^III^ coordination is attributed to energy transfer to the ^5^D_0_ level of the Eu^III^ ion, which can be assumed to quench the ^3^MLCT states (Table 2).

From the emission spectra and the ^5^D_0_ emission lifetime of the Eu^III^ ion, the radiative (A_r_) and nonradiative (A_nr_) decay rates, the intrinsic Eu^III^ emission quantum yield (${\Phi}_{Eu}^{Eu}$), the Judd–Ofelt intensity parameters (Ω_λ_), and the intensity ratio of the ^5^D_0_ *→* ^7^F_2_/^5^D_0_ *→* ^7^F_1_ (R_0–2/0–1_) transitions were determined for each solvent investigated. To this end, time-resolved emission spectra of the Ir^III^-Eu^III^ complex were acquired to suppress the residual emission from the Ir^III^-p ligand complex, thereby eliminating its interference in the determination of the Eu^III^ photoluminescence parameters. The results are summarized in Table 2 and Table S9. These parameters were calculated using the set of equations provided in Section II of the Supporting Information.

The radiative decay rate (A_r_) is nearly identical across all three solvents, indicating that solvent polarity has minimal impact on the radiative pathway and supporting the conclusion that the ligand environment predominantly governs sensitization. In contrast, the nonradiative decay rates (A_nr_) in acetonitrile and DMSO are approximately half of those observed in ethyl acetate, consistent with the higher coordination ability of these solvents. This behavior likely results from the substitution of water molecules in the Eu^III^ coordination sphere by solvent molecules, thereby reducing vibronic quenching of the Eu^III^ emission [39]. This trend is reflected in the intrinsic Eu^III^ emission quantum yield (${\Phi}_{Eu}^{Eu}$), further confirming the role of solvent coordination in mitigating nonradiative deactivation pathways. Since minimal or no residual emission from the Ir^III^ moiety was observed in these solvents, the sensitization efficiencies^2^ were calculated using the following equation: η = *Φ*/${\Phi}_{Eu}^{Eu}$, yielding values of 67, 81, and 89% for ethyl acetate, DMSO, and acetonitrile, respectively. This trend reinforces that increasing solvent polarity enhances energy transfer efficiency to the Eu^III^ ion.

Structural distortions within the Eu^III^ coordination environment can be further elucidated using Judd–Ofelt parameters. Notably, the Ω_2_ parameter (Table S9) shows decreases in DMSO relative to ethyl acetate and acetonitrile, suggesting a higher symmetry around the Eu^III^ site, likely due to the coordination of symmetric DMSO molecules. This behavior is corroborated by the spectral area ratio between the ^5^D_0_ → ^7^F_2_ and ^5^D_0_ → ^7^F_1_ transitions. While the ^5^D_0_ → ^7^F_1_ transition rate, allowed via a magnetic dipole mechanism, is relatively insensitive to the surrounding electric field, the ^5^D_0_ → ^7^F_2_ transition rate is highly sensitive to changes in the local electric environment. As a result, subtle distortions in the coordination sphere can modulate its spontaneous decay rate [40]. The observed decrease in the R_(0–2)/(0–1)_ thus confirms a higher symmetry around Eu^III^ in DMSO [37].

Aprotic polar solvents play a crucial role in the Eu^III^ sensitization process, even when the donor ^3^MLCT state is close in energy to the emissive ^5^D_0_ state of the Eu^III^. Additionally, the coordination ability of the solvent molecules can displace quenching water molecules from the Eu^III^ coordination sphere, reducing vibronic coupling via OH oscillators — a known pathway for emission quenching that will be discussed further in the next sub-section. This effect, combined with the solvent polarity, helps rationalize the observed variations in emission lifetimes, quantum yields, and Judd–Ofelt parameters across different solvents.

#### Protic Polar Solvents (Polarity > 1.7 D)

**Fig. 5:**
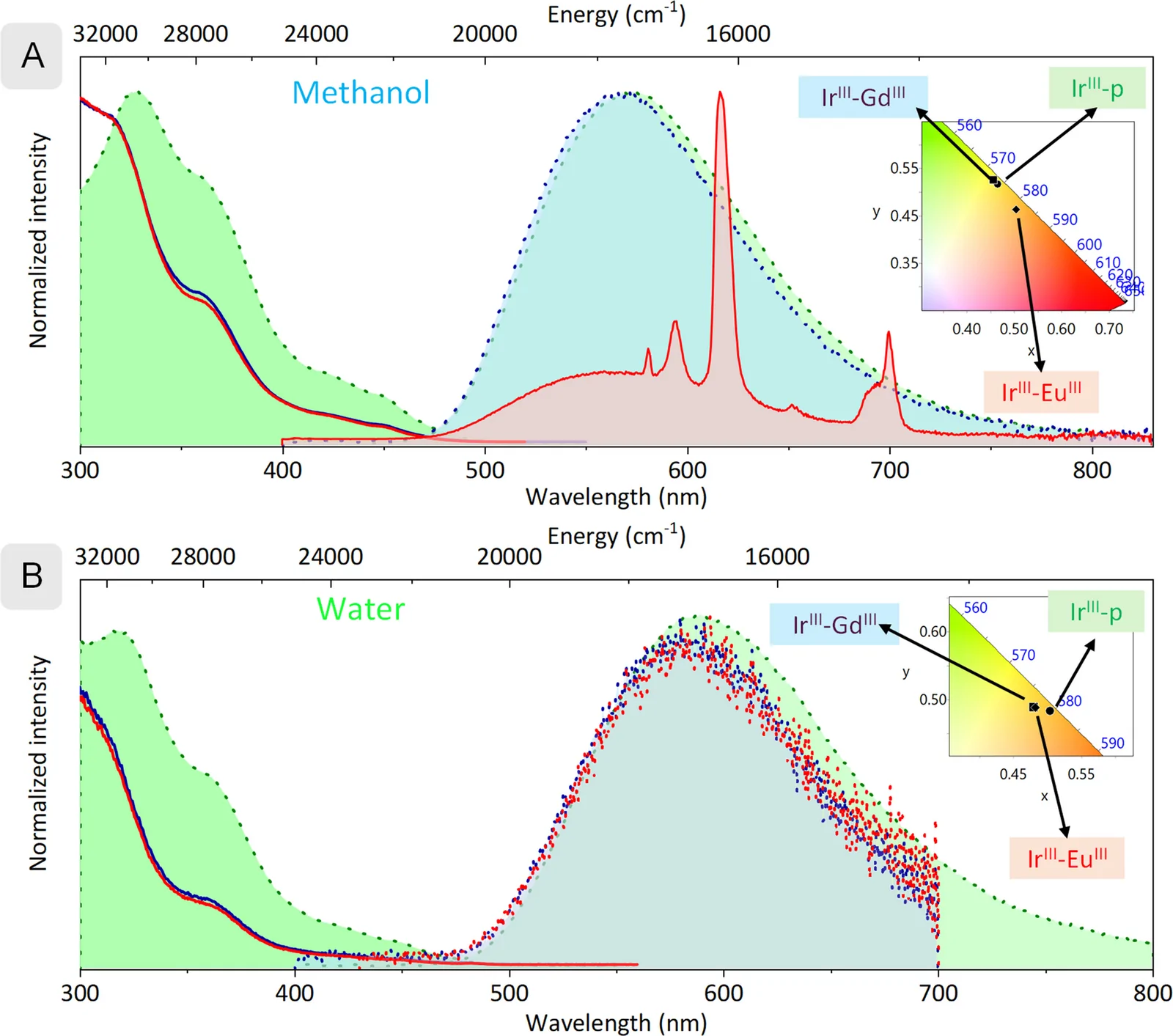
Excitation and emission spectra of Ir^III^-p (green), Ir^III^-Gd^III^ (blue), and Ir^III^-Eu^III^ (red) complexes measured in **A**) methanol and **B**) water. Concentration in methanolic solution of 1.0 × 10^–5^ mol·L^−1^. All measurements were carried out with a bandpass of 2.5 nm for both Ex and Em, with a 0.5 nm increment, an integration time of 0.5 s, and excitation at 316 nm

In contrast to aprotic polar solvents, protic polar solvents such as methanol and water lead to pronounced luminescence quenching due to the presence of OH oscillators, which promote an efficient nonradiative deactivation pathway. Although the excitation spectra remain largely unaffected, the emission spectra display significant differences. In methanol, the broad ^3^MLCT emission from the Ir^III^ moiety becomes relatively more pronounced compared to the narrow *f*-*f* emission bands of Eu^III^, indicating suppression of the Eu^III^ luminescence. In water, however, the Eu^III^ *f*-*f* emission is completely quenched, as shown in Fig. 5, confirming that protic solvents open an efficient nonradiative mechanism via vibronic coupling. If, on the one hand, the emission from the Eu^III^ is quenched, the emission from the Ir^III^ moiety, on the other hand, seems much less affected.

First, it is important to note that, similarly to what is observed in aprotic polar solvents, the ^3^MLCT emission in the bimetallic complexes also undergoes a slight blueshift compared to the Ir^III^ complex. Specifically, the ^3^MLCT emission shifts from 17452 cm^−1^ (573 nm) to 17637 cm^−1^ (567 nm) in methanol, and from 17006 cm^−1^ (588 nm) to 17271 cm^−1^ (579 nm) in water (Figure S5 and Table 1). However, despite these minor energy shifts and the relatively large energy difference between the donor and acceptor states in methanol (e.g., 410 cm^−1^, Table 1), the high polarity of these protic solvents (methanol, 1.70 D; water, 1.85 D) does not promote efficient Eu^III^ sensitization. This demonstrates that, in contrast to aprotic polar solvents, the presence of OH oscillators in protic media strongly enhances nonradiative deactivation pathways, leading to substantial quenching of the Eu^III^ emission even when the donor–acceptor energy gap efficiently populates the excited states of the Eu^III^.

By comparing the emission quantum yield and lifetime of the Ir^III^ complex with those of the Ir^III^-Gd^III^ bimetallic complex, an increase in both parameters is observed, more pronounced in methanolic solution than in aqueous solution, as shown in Table 2. In aqueous solution, the complexes required super-dilute conditions for measurement due to their low solubility, which would otherwise cause light-scattering effects. Interestingly, in methanol, both the emission quantum yield and lifetime of the Ir^III^-Gd^III^ complex are slightly higher than those measured in aprotic polar solvents, suggesting that OH oscillators exert minimal influence on the ^3^MLCT emissive state. In contrast, the behavior of the Ir^III^-Eu^III^ bimetallic complex is markedly different: in protic solvents, both the overall and intrinsic emission quantum yields decrease significantly, reflecting the strong quenching effect of OH oscillators on the Eu^III^ emission, consistent with the trends observed in the previously discussed emission spectra.

It is well established that aqueous solutions provide an efficient quenching pathway for many Eu^III^ complexes due to vibronic coupling between high-frequency OH oscillators and the excited ^5^D_0_ level of Eu^III^ [41]. The efficiency of vibronic coupling quenching depends on the number of vibrational overtones required to connect the energy gap between the ^5^D_0_ emission level and the nearest fundamental level (^7^F_6_). The fewer the required overtones, the more effective the nonradiative deactivation. Considering that the fundamental OH vibration energy is approximately 3450 cm^−1^ and the energy gap between the ^5^D_0_ and ^7^F_6_ levels is ~ 12300 cm^−1^, the quenching occurs primarily via the 3rd or 4th OH vibrational overtone [42]. This accounts for the high nonradiative decay rate (A_nr_) observed in methanolic solutions and corroborates the trends observed in protic solvents, where OH oscillators significantly decrease both the intrinsic and overall emission quantum yields of Eu^III^. Figure S9 schematically illustrates this vibrational coupling between the ^5^D_0_ level and some typical oscillators. When water molecules are coordinated to the Eu^III^ ion, nonradiative deactivation via OH oscillators becomes so efficient that the *f*-*f* emission of the Eu^III^ center in the Ir^III^-Eu^III^ complex is completely quenched. As a result, photoluminescent parameters could not be determined in aqueous solution, underscoring the dominant quenching effect of coordinated water. In methanolic solution, however, the Eu^III^ emission is partially retained, enabling the calculation of Judd–Ofelt parameters (Table S10). Compared to aprotic polar solvents, the Ω_2_ parameter reveals significant distortion in the Eu^III^ coordination sphere. In contrast, the Ω_4_ parameter suggests higher polarizability around the Eu^III^ ion, probably due to a reduced distance between the Eu^III^ ion and its coordinating atoms compared to aprotic environments.

After analyzing the photophysical properties in polar protic solvents, such as methanol and water, these solvents are not suitable media for investigating the photophysical properties of bimetallic Eu^III^ complexes because their OH groups can coordinate to the Eu^III^ ion, thereby providing highly efficient nonradiative quenching pathways. In these environments, solvent polarity is insufficient to compensate for the relatively small energy gap between the donor (^3^MLCT) state and acceptor (^5^D_0_) level. Consequently, emission from the Ir^III^ unit becomes predominant, while the characteristic Eu^III^ *f*-*f* emission is significantly reduced or fully quenched, particularly in aqueous solution. This behavior underscores the crucial role of solvent coordination and vibrational deactivation in modulating the luminescent properties of Eu^III^-based bimetallic complexes.

#### Solvents With Dipole Momentum Lower Than 1.7 D

In solvents with dipole momentum lower than 1.7 D such as dichloromethane and chloroform, the emission band of the Ir^III^-Gd^III^ bimetallic complex displays a pronounced blueshift relative to the Ir^III^-p, consistent with the trends observed in aprotic and protic polar solvents. In dichloromethane, the maximum emission shifts from 17271 cm^−1^ (579 nm) to 17921 cm^−1^ (558 nm), whereas in chloroform it shifts from 17422 cm^−1^ (574 nm) to 17986 cm^−1^ (556 nm) (Figure S5). The excitation spectra exhibit minimal changes across these solvents, indicating that the absorption characteristics of the Ir^III^ chromophore are largely insensitive to solvent polarity.

**Fig. 6:**
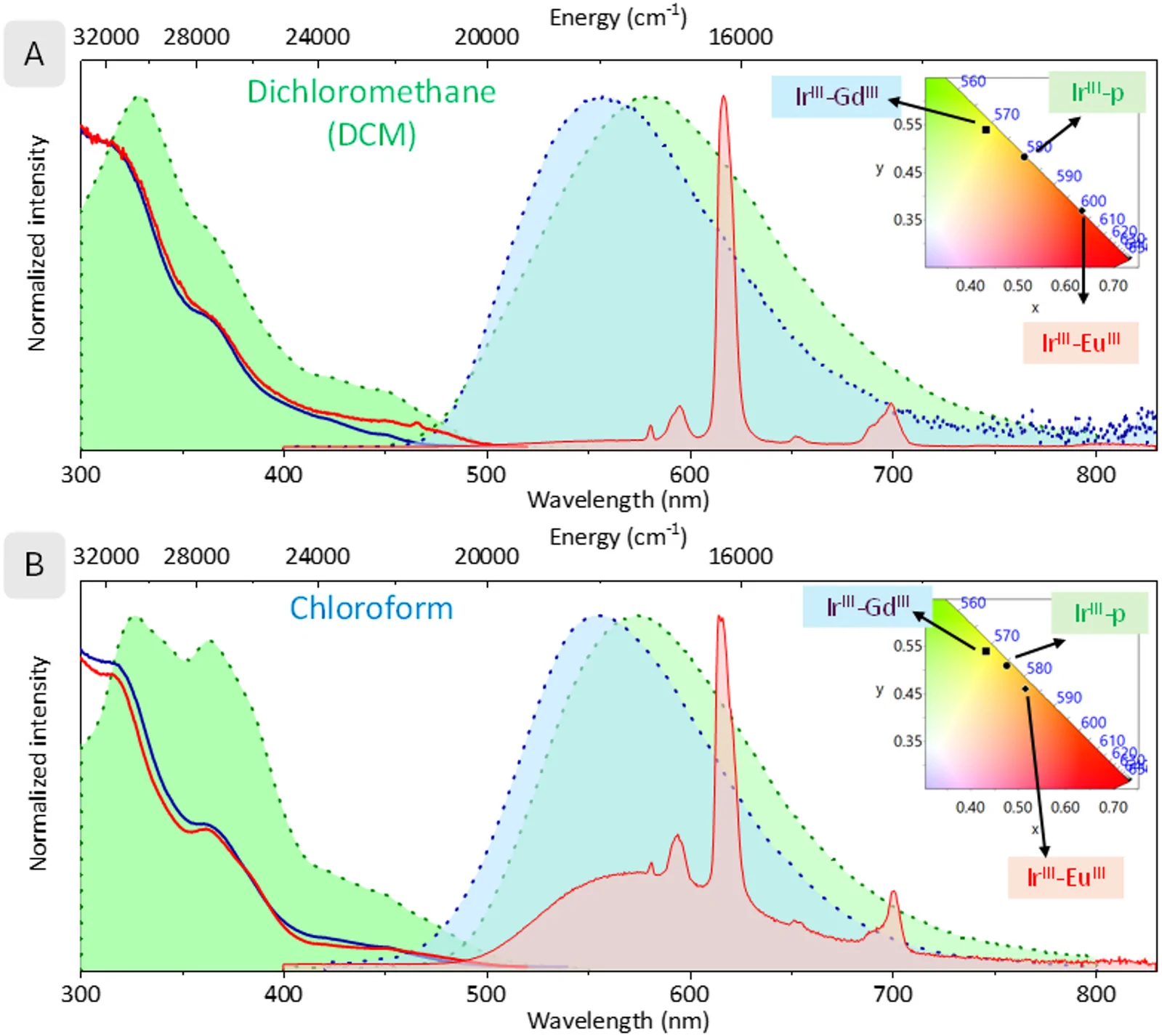
Excitation and emission spectra of Ir^III^-p (green), Ir^III^-Gd^III^ (blue), and Ir^III^-Eu^III^ (red) complexes measured in **A**) dichloromethane and **B**) chloroform. Concentration in solution of 1.0 × 10^–5^ mol·L^−1^. All measurements were carried out with a bandpass of 2.5 nm for both Ex and Em, with a 0.5 nm increment and an integration time of 0.5 s

The sensitization of the Eu^III^ ion, however, is strongly influenced by the energy difference between the ^3^MLCT donor state and the ^5^D_0_ acceptor state, as illustrated by the emission spectra in Fig. 6. In dichloromethane, the high-energy ^3^MLCT state (17921 cm^−1^) enables efficient energy transfer, resulting in emission predominantly characterized by the narrow Eu^III^ bands in the orange-red region. In contrast, in chloroform solution, the ^3^MLCT donor state (17986 cm^−1^) shows an energy similar to that observed in dichloromethane; however, inefficient sensitization of the Eu^III^ ion is observed. Unlike acetonitrile, DMSO, ethyl acetate, or even dichloromethane, chloroform's low polarity (1.04 D) does not promote efficient energy transfer, resulting in a dual-emission profile, as shown in Fig. 6.

By comparing the emission quantum yields and lifetimes of the Ir^III^-p complex with those of the bimetallic complexes in less polar solvents, a consistent trend emerges (Table 2). The bimetallic complexes exhibit increased emission quantum yields and longer lifetimes. However, for the Ir^III^-Eu^III^ complex, the overall emission quantum yields were lower than those observed for the Ir^III^-Gd^III^ complex. This reduction can be primarily attributed to the limited coordinating ability of the low-polar solvents, which allows water molecules to remain bound to the Eu^III^ ion and act as quenchers via vibronic coupling, as illustrated schematically in Figure S9. This phenomenon parallels the behavior observed in ethyl acetate, where the nonradiative decay rates (A_nr_) are similar: 777 s^−1^ in ethyl acetate and 785 s^−1^ in dichloromethane, suggesting a comparable degree of quenching in both media.

The Judd–Ofelt analysis for the Eu^III^ ion in solvents with dipole momentum lower than 1.7 D reveals that the Ω_2_ values indicate a relatively more symmetrical coordination environment compared to that observed in aprotic polar solvents, while being comparable to the symmetry found in protic polar solvents. This suggests that, despite the lower polarity of these solvents, the Eu^III^ ion exhibits reduced coordination distortion compared with its behavior in aprotic polar solvents. In contrast, the Ω_4_ values point to an increase in the Eu^III^···ligand bonding distance in solvents with lower polarities, consistent with a looser coordination environment. These results are summarized in Table S11.

### Antenna Pathway and Efficiency

Two primary mechanisms are generally considered for energy transfer in *d-f* heterobimetallic complexes: multipolar and exchange energy transfer, both of which exhibit strong distance dependence.^17^ The multipolar mechanism [43] relies on spectral overlap between donor emission and acceptor absorption, and the efficiency follows an inverse sixth-power distance dependence (d^−6^) for dipole–dipole interactions, an eighth-power distance dependence (d^−8^) for dipole-quadrupole, and a tenth-power distance dependence (d^−10^) for quadrupole–quadrupole [44]. The exchange^43^ transfer requires orbital overlap between donor and acceptor, and the efficiency decays exponentially with donor–acceptor distance. In *d–f* assemblies containing conjugated bridging ligands, however, efficient long-range exchange-type transfer may occur via a super-exchange pathway, allowing energy migration over distances approaching 20 Å while maintaining its characteristic exponential distance dependence [45].

These considerations highlight the importance of the donor–acceptor separation in determining the efficiency and mechanism of energy transfer. Since single crystals suitable for X-ray diffraction could not be obtained for the Ir^III^–Eu^III^ complex, the Ir^III^···K^I^ distances determined from the crystal structure of the corresponding Ir^III^ precursor may serve as an approximate estimate of the donor–acceptor distance (Ir^III^···Eu^III^), which ranges from 8.418 to 8.458 Å. It should be noted that the donor–acceptor distance is not necessarily equivalent to the Ir^III^···Eu^III^ separation, since the donor excited state possesses significant ^3^MLCT character, resulting in a substantial contribution of ligand-centered orbitals to the energy-transfer process. The relatively short donor–acceptor separation found for this system is consistent with exchange-mediated transfer reported for similar systems in the literature [17, 46, 47], yet we cannot exclude that other mechanisms may contribute to the energy transfer.

To elucidate the Ir^III^ → Eu^III^ apparent energy-transfer rate constants (k_ET_) in all tested solvents, the following equation was used [48]:

1 $${k}_{ET}= \frac{1}{{\tau}_{q}}-\frac{1}{{\tau}_{u}}$$

where τ_q_ is the residual lifetime of the Ir^III^-based complex used as a ligand undergoing quenching by europium ion, and τ_u_ is the “unquenched” lifetime of the reference complex, Ir^III^-Gd^III^. The significant decrease in the ^3^MLCT emission lifetime of the Ir^III^-Eu^III^ complex compared to its Gd^III^ analog can be attributed to ET quenching from the metalloligand to the bound Eu^III^ ions. The values obtained in Table 3 indicate a tendency toward higher apparent energy-transfer rate constants in more polar solvents, consistent with the trends observed in the emission spectra discussed above. However, the energy mismatch between the donor and acceptor states must also be considered, as it plays a critical role in determining the transfer efficiency. Another important point to note is that higher *k*_ET_ values are observed in solvents with greater coordinating ability and lacking O–H oscillators, underscoring that these factors may also influence the energy transfer process. Therefore, these results suggest that both solvent-dependent effects and the donor–acceptor energy gap contribute to modulating energy transfer.

**Table 3:** ^3^MLCT residual emission lifetime (τ_q_) obtained from the Ir^III^-Eu^III^ complex, ^3^MLCT emission lifetime (τ_u_) obtained from the Ir^III^-Gd^III^ complex, apparent energy-transfer rate constants (k_ET_), and donor-to-acceptor energy-transfer efficiency (η_ET_) in the Ir^III^-Eu^III^ complex

	τ (ns)		*k*_*ET*_ (s^−1^)	*η*_*ET*_ (%)
*Ethyl Ac*	Ir^III^-Eu^III^	47.5	1.7 × 10^7^	82
	Ir^III^-Gd^III^	265.2		
*ACN*	Ir^III^-Eu^III^	163.2	4.3 × 10^6^	71
	Ir^III^-Gd^III^	557.8		
*DMSO*	Ir^III^-Eu^III^	35.3	2.6 × 10^7^	90
	Ir^III^-Gd^III^	368.0		
*Methanol*	Ir^III^-Eu^III^	232.6	2.3 × 10^6^	54
	Ir^III^-Gd^III^	505.3		
*Water*	Ir^III^-Eu^III^	103.5	5.5 × 10^6^	57
	Ir^III^-Gd^III^	240.3		
*DCM*	Ir^III^-Eu^III^	176.5	4.1 × 10^6^	72
	Ir^III^-Gd^III^	637.2		
*DCM (degassed)*	Ir^III^-Eu^III^	182.3	4.0 × 10^6^	73
	Ir^III^-Gd^III^	678.7		
*Chloroform*	Ir^III^-Eu^III^	431.1	1.0 × 10^6^	43
	Ir^III^-Gd^III^	760.7		
*Chloroform (degassed)*	Ir^III^-Eu^III^	529.1	8.2 × 10^5^	44
	Ir^III^-Gd^III^	934.8		

To assess the donor-to-acceptor energy-transfer efficiency (η_ET_), the residual ^3^MLCT emission lifetime (${\tau}_{q}$) of the Ir^III^-Eu^III^ and the ^3^MLCT emission lifetime (${\tau}_{u}$) of the corresponding Ir^III^–Gd^III^ complex were employed in the following expression [47]:

2 $${\eta}_{ET}= 1-\frac{{\tau}_{q}}{{\tau}_{u}}$$

The results reveal a tendency toward higher donor-to-acceptor energy-transfer efficiency in more polar solvents, in agreement with the apparent energy-transfer rate constants (k_ET_) and the spectroscopic observations discussed above. Water and methanol, however, display a different behavior. Although moderate donor-to-acceptor energy-transfer efficiency is observed in these solvents, the high concentration of O–H oscillators strongly promotes non-radiative relaxation pathways, resulting in substantial quenching of the Eu^III^ emission. For aqueous solutions, this quenching is sufficiently efficient to completely suppress the Eu^III^-centered emission despite significant energy transfer. Both k_ET_ and η_ET_ were evaluated for degassed DCM and chloroform solutions. While the removal of dissolved oxygen increased the ^3^MLCT emission lifetime and slightly affected the k_ET_ values, only a minor enhancement in donor-to-acceptor energy-transfer efficiency was observed, indicating that oxygen quenching has a limited influence on the overall donor-to-acceptor energy-transfer process.

In summary, the photophysical behavior of Ir^III^-Eu^III^ bimetallic complexes in solution is strongly modulated by the properties of the solvent. The following key points can be raised:

Polar solvents facilitate energy transfer: Highly polar solvents promote Eu^III^ sensitization by stabilizing the excited states and compensating for relatively small donor–acceptor energy gaps.

Donor state energy governs sensitization in low-polarity media: In non-polar or low-polarity solvents, the efficiency of Eu^III^ sensitization is primarily dictated by the energy of the ^3^MLCT donor state, reflecting behavior like that observed in the solid state.

Solvent coordination affects emission efficiency: Non-coordinating solvents moderately enhance emission quantum yields by reducing access of quenching species to the Eu^III^ ion. Coordinating solvents lacking OH oscillators further improve both overall and intrinsic emission quantum yields by displacing water molecules and other potential quenchers.

Protic solvents act as strong quenchers: Solvents containing OH oscillators, such as methanol and water, induce strong vibronic quenching of Eu^III^ emission, resulting in significantly reduced emission quantum yields and shorter lifetimes.

These rationalizations, which correlate solvent polarity, coordinating ability, presence of OH oscillator groups, and the observed overall emission quantum yields, are summarized in Table 4.

**Table 4:** Solvent properties and influence on the photoluminescent properties of the Ir^III^-Eu^III^ bimetallic complex studied in this work

	Polarity	Energy of ^3^MLCT	Coordinating	OH oscillators	Energy transfer to Eu^III^	*Φ* of Ir^III^-Eu^III^
*Ethyl Ac*	Moderate	Moderate	No	No	Favorable	Moderate
*ACN*	High	Low	Yes	No	Favorable	High
*DMSO*	High	Low	Yes	No	Favorable	High
*Methanol*	Moderate	Moderate	Yes	Yes	Partial	Low
*Water*	Moderate	Low	Yes	Yes	No favorable	Low
*DCM*	Moderate	Moderate	No	No	Favorable	Moderate
*Chloroform*	Low	Moderate	No	No	Partial	Moderate

* Low polarity: < 1.5 D; moderate polarity > 1.5 D and < 1.9 D; and high polarity > 1.9 D

## Conclusions

The photophysical behavior of Ir^III^-Ln^III^ bimetallic complexes (Ln^III^ = Eu^III^ or Gd^III^) in solution is strongly modulated by the interplay among the donor–acceptor energy gap, solvent polarity, and solvent coordination ability. In aprotic polar solvents, efficient Eu^III^ sensitization occurs even when the ^3^MLCT donor state lies close in energy to the Eu^III 5^D_0_ level, due to the ability of highly polar solvents to stabilize excited states and displace quenching water molecules from the coordination sphere. Systematic blueshifts in the ^3^MLCT emission reveal that less polar solvents exert a minimal influence on Ir^III^ excited states, while more polar solvents induce stabilization and observable redshifts. Additionally, bimetallic complexes exhibit higher emission quantum yields and longer lifetimes than Ir^III^-p, owing to enhanced structural rigidity and reduced nonradiative decay. Judd–Ofelt analysis reveals that solvent coordination modulates the symmetry and bonding environment of the Eu^III^ ion, thereby influencing both the hypersensitive ^5^D_0_ → ^7^F_2_ transition and intrinsic emission quantum yields. In contrast, polar protic solvents such as methanol and water strongly quench Eu^III^ emission via vibronic coupling with coordinated OH oscillators. In low-polarity or non-coordinating solvents, sensitization efficiency is primarily governed by the ^3^MLCT donor-state energy, whereas coordinating solvents without OH oscillators maximize emission by preventing quenching species from accessing the lanthanide ion. The apparent Ir^III^ → Eu^III^ energy-transfer rate constants (k_ET_) and donor-to-acceptor energy-transfer efficiencies (η_ET_) were estimated from the residual ^3^MLCT emission lifetimes (τ_q_) of the Ir^III^–Eu^III^ complex and the corresponding Ir^III^–Gd^III^ analog (τ_u_). The results also showed a general tendency toward higher energy-transfer rates and efficiencies in more polar solvents. Degassing experiments further demonstrated that dissolved oxygen exerts only a limited influence on the overall sensitization process. Overall, the results demonstrate that both solvent polarity and coordination properties are critical factors for tuning Eu^III^ sensitization and optimizing luminescence in Ir^III^-Eu^III^ bimetallic complexes. These findings provide valuable guidelines for the rational design of highly emissive lanthanide-based materials in solutions.

## Supplementary Information

Below is the link to the electronic supplementary material.

Supplementary file1 (PDF 1.50 MB)

## Data Availability

The datasets generated and analyzed during the current study are available from the corresponding author on reasonable request. Relevant data supporting the findings of this study are also included in the article and its supplementary information files.
