# Supplementary material for: Solvent-dependent Sensitization and Photophysical Behavior of IrIII-EuIII Bimetallic Complexes
Source: J Fluoresc. 2026 Aug 11;36(9):5307–22. doi: 10.1007/s10895-026-04895-1 (PMC13615140; doi:10.1007/s10895-026-04895-1)
Supplement: Supplementary file 1 [file 10895_2026_4895_MOESM1_ESM.pdf]

Supplementary Information

Solvent-Dependent Sensitization and Photophysical Behavior of Ir^{III}-Eu^{III} Bimetallic Complexes

Felipe S. M. Canisares,[a,b] Vytor C. Oliveira,[d,e] Alessandro B. S. Garcia,[a] João Honorato,[b] Javier A. Ellena,[c] Marian R. Davolos,[a] Ana M. Pires,[a,d,e] Sergio A. M. Lima[a,d,e].

[a] São Paulo State University (UNESP), Institute of Chemistry, Araraquara, SP, Brazil.

[b] University of São Paulo (USP), Institute of Chemistry, São Paulo, SP, Brazil

[c] University of São Paulo (USP), Institute of Physics, São Carlos, SP, Brazil

[d] São Paulo State University (UNESP), School of Technology and Sciences, Presidente Prudente, SP, Brazil.

[e] São Paulo State University (UNESP), Institute of Biosciences, Humanities and Exact Sciences, São José do Rio Preto, SP, Brazil.

Corresponding Author: Sergio Antonio Marques Lima

São Paulo State University (UNESP), School of Technology and Sciences, Department of Chemistry and Biochemistry, R. Roberto Simonsen, 305, 19060-900 – Presidente Prudente, São Paulo, Brazil.

Corresponding Author email address: sergio.lima@unesp.br

KEYWORDS: Iridium, Europium, Bimetallic complexes, Solvent effects, Energy transfer, Photoluminescence, Judd–Ofelt analysis.

FIGURE LIST

Figure S1. Crystal structure of Ir^{III}-p. 20

Figure S2. A) FTIR spectra of the Ir^{III}p, Ir^{III}-Gd^{III}, and Ir^{III}-Eu^{III} complexes, B) magnification of the region from 1,350 cm^{-1} to 1,650 cm^{-1}. 20

Figure S3. A) FTIR spectra of the Na_2bpdc ligand, Ir^{III}-Gd^{III}, and Ir^{III}-Eu^{III} complexes; and B) most common carboxylate coordination modes and the energy difference between vass(COO^-) and vsym(COO^-) for each coordination mode in the complexes (Δv_c) compared with the energy difference in the ligands (Δv_L). To illustrate, it was used a generic carboxylic acid (benzoic acid). 21

Figure S4. A) emission spectra obtained after each addition of $EuCl_3$ and B) the graph of the ratio of Ir^{III}/Eu^{III} and the energy of the maximum emission of the Ir^{III} complex. C) emission spectra obtained after each addition of $GdCl_3$ and D) the graph of the ratio of Ir^{III}/Gd^{III} and the energy of the maximum emission of the Ir^{III} complex. 22

Figure S5. Excitation and emission spectra of A) Ir^{III}-p; B) Ir^{III}-Gd^{III}; and C) Ir^{III}-Eu^{III} complexes in the solid state and in various solvents. Concentration in solution of 1.0x10^{-5} mol·L^{-1}. All measurements were carried out with a bandpass of 2.5 nm for both Ex and Em, with an increment of 0.5 nm and an integration time of 0.5 s. Some Ir^{III}-Gd^{III} emission spectra (chloroform, ethyl acetate, and acetonitrile) are contaminated with Eu^{III} ion, as can be seen by the appearance of a narrow emission band at approximately 616 nm, what has no interference in the energy determination of the emissive triplet state. 23

Figure S6. Excitation spectra of Ir^{III}-Eu^{III} complex in various solvents. Concentration in solution of 1.0x10^{-5} mol·L^{-1}. All measurements were carried out with a bandpass of 2.5 nm for both Ex and Em, with a 0.5 nm increment and an integration time of 0.5 s. 24

Figure S7. Lippert-Mataga plot for Ir^{III}-p bimetallic complex. 24

Figure S8. Lippert-Mataga plot for Ir^{III}-Gd^{III} bimetallic complex. 25

Figure S9. Schematic representation of vibrational coupling between the $^5D_0$ emitting level of Eu^{III} and selected common oscillators. The energy values are given in cm^{-1}. A generic Eu^{III} coordination sphere is included to illustrate the spatial arrangement of OH oscillators responsible for nonradiative deactivation. Adapted from []. 26

TABLE LIST

Table S1. Crystallographic Data for Ir^{III}-p. 4

Table S2. Fractional Atomic Coordinates ($\times10^4$) and Equivalent Isotropic Displacement Parameters ($Å^2\times10^3$) for Ir^{III}-p complex. U_{eq} is defined as 1/3 of the trace of the orthogonalized U_{IJ} tensor. 4

Table S3. Anisotropic Displacement Parameters ($Å^2\times10^3$) for Ir^{III}-p. The Anisotropic displacement factor exponent takes the form: $-2\pi^2[h^2a^{*2}U_{11}+2hka^*b^*U_{12}+\ldots]$. 7

Table S4. Bond Lengths for Ir^{III}-p. 10

Table S5. Bond Angles for Ir^{III}-p. 11

Table S6. Torsion Angles for Ir^{III}-p. 15

Table S7. Hydrogen Atom Coordinates ($Å\times10^4$) and Isotropic Displacement Parameters ($Å^2\times10^3$) for Ir^{III}-p. 18

Table S8. Solvent masks information for Ir^{III}-p. 19

Table S9. Judd-Ofelt parameters (Ω_2 and Ω_4) and area ratio between emission bands related to $^5D_0\rightarrow{}^7F_2$ and $^5D_0\rightarrow{}^7F_1$ transitions. 25

Table S10. Judd-Ofelt parameters (Ω_2 and Ω_4) and area ratio between emission bands related to $^5D_0\rightarrow{}^7F_2$ and $^5D_0\rightarrow{}^7F_1$ transitions. 26

Table S11. Judd-Ofelt parameters (Ω_2 and Ω_4) and area ratio between emission bands related to $^5D_0\rightarrow{}^7F_2$ and $^5D_0\rightarrow{}^7F_1$ transitions. 26

SUPLEMENTARY INFORMATION – SECTION I

Table S1. Crystallographic Data for Ir^{III}-p.

	Ir^{III}-p
**CCDC code**	2525129
**Empirical formula**	$C_{34}H_{50}F_4IrK_{0.5}N_4O_{20}$
**Formula weight**	1122.550
**temperature, K**	104(6)
**wavelength (Å)**	1.54184
**crystal system**	Triclinic
**space group**	*P-1*
**a (Å)**	12.0261(1)
**b (Å)**	17.8871(3)
**c (Å)**	23.2310(3)
**α (deg)**	69.922(2)
**β (deg)**	88.969(1)
**γ (deg)**	75.639(1)
**volume (Å^3)**	4534.50(12)
**Z**	4
**crystal size**	0.136 × 0.091 × 0.017
**density, calcd (g/cm^3)**	1.644
**Radiation**	Cu Kα (λ = 1.54184)
**absorption coefficient (mm^{-1})**	6.842
**F(000)**	2251.1
**2Θ range for data collection (deg)**	8.88 to 140.14
**Index ranges**	-12 ≤ h ≤ 15, -22 ≤ k ≤ 22, -29 ≤ l ≤ 29
**reflections collected**	85614
**independent reflections**	17099 [R_{int} = 0.0527, R_{sigma} = 0.0422]
**data/restraints/parameters**	17099/72/951
**goodness-of-fit on F^2**	0.986
**Final R indexes [I>=2σ (I)]**	R_1 = 0.0319, wR_2 = 0.0828
**Final R indexes [all data]**	R_1 = 0.0381, wR_2 = 0.0855
**Largest diff. peak/hole (e Å^{-3})**	1.42/-0.65

Table S2. Fractional Atomic Coordinates (×10^4) and Equivalent Isotropic Displacement Parameters (Å^2×10^3) for Ir^{III}-p complex. U_{eq} is defined as 1/3 of the trace of the orthogonalized U_{IJ} tensor.

Atom	*x*	*Y*	*z*	U(eq)
**Ir1a**	**-183.8(3)**	**6375.7(2)**	**1394.87(15)**	**41.86(18)**
**Ir1b**	**3788.6(3)**	**9216.5(2)**	**2212.47(18)**	**49.61(19)**
**K1**	**2472.9(12)**	**5115.7(11)**	**5028.4(7)**	**42.3(5)**
**F1a**	**2950(3)**	**6190(2)**	**-280.9(15)**	**92.8(10)**
**F1b**	**823(3)**	**10626.2(18)**	**188.9(14)**	**93.7(10)**
**F2a**	**2001(2)**	**8585.4(16)**	**197.6(13)**	**72.2(7)**
**F2b**	**404(4)**	**11906(2)**	**1643(2)**	**135.8(15)**
**F3a**	**-3303(2)**	**8479.8(17)**	**-459.9(11)**	**73.0(7)**
**F3b**	**5911(4)**	**11661(2)**	**1245.4(18)**	**114.4(12)**

**F4a**	**-2358(3)**	**5704(2)**	**-239.6(14)**	**95.7(10)**
**F4b**	**6431(3)**	**9627(2)**	**357.2(14)**	**90.2(9)**
**O1a**	**1066(2)**	**5838.3(19)**	**4263.7(11)**	**58.3(7)**
**O1b**	**3185(2)**	**6255.0(18)**	**4754.2(13)**	**64.6(8)**
**O1w**	**3984(2)**	**4577(2)**	**4482.0(14)**	**67.1(8)**
**O2a**	**-613.3(19)**	**6374.4(18)**	**3727.0(12)**	**55.7(7)**
**O2b**	**4565(2)**	**6903.9(19)**	**4622.4(12)**	**62.0(7)**
**O2w**	**1559(2)**	**4045(2)**	**5097.7(14)**	**67.8(8)**
**O3a**	**486(2)**	**4403.1(18)**	**3899.1(12)**	**60.6(7)**
**O3b**	**4547.7(19)**	**5965.4(16)**	**3592.5(13)**	**55.3(7)**
**O4a**	**-1213(2)**	**4578(2)**	**4279.3(12)**	**65.5(8)**
**O4b**	**6164(2)**	**5533.0(17)**	**4174.8(12)**	**58.8(7)**
**N1a**	**970(2)**	**5961(2)**	**2188.6(13)**	**47.5(7)**
**N1b**	**5108(2)**	**8332.6(19)**	**2861.6(14)**	**49.7(7)**
**N2a**	**-1217(2)**	**5992.2(19)**	**2141.5(13)**	**45.2(7)**
**N2b**	**2903(2)**	**8417(2)**	**2836.8(14)**	**51.3(8)**
**N3a**	**-336(2)**	**7527.0(18)**	**1417.3(12)**	**43.7(6)**
**N3b**	**3282(3)**	**9997(3)**	**2676.9(17)**	**70.5(9)**
**N4a**	**-41(3)**	**5270(2)**	**1290.2(15)**	**54.3(8)**
**N4b**	**4268(2)**	**8510.7(19)**	**1671.5(13)**	**47.7(7)**
**C1a**	**2064(3)**	**6023(3)**	**2153.4(17)**	**52.0(9)**
**C1b**	**6233(3)**	**8327(3)**	**2809.7(19)**	**59.5(11)**
**C2a**	**2690(3)**	**6015(3)**	**2646.8(17)**	**54.2(9)**
**C2b**	**7097(3)**	**7656(3)**	**3110.3(19)**	**59.9(11)**
**C3a**	**2150(3)**	**6005(2)**	**3173.7(16)**	**50.1(9)**
**C3b**	**6812(3)**	**6930(2)**	**3441.5(16)**	**51.0(9)**
**C4a**	**1012(3)**	**5940(2)**	**3223.0(15)**	**45.1(8)**
**C4b**	**5662(3)**	**6910(2)**	**3501.1(15)**	**47.1(8)**
**C5a**	**467(3)**	**5863(2)**	**2730.1(14)**	**40.8(7)**
**C5b**	**4820(3)**	**7654(2)**	**3240.1(15)**	**44.4(8)**
**C6a**	**-686(3)**	**5681(2)**	**2715.8(15)**	**43.3(8)**
**C6b**	**3556(3)**	**7798(2)**	**3314.6(16)**	**46.8(8)**
**C7a**	**-1173(3)**	**5200(2)**	**3208.3(16)**	**46.2(8)**
**C7b**	**3033(3)**	**7390(2)**	**3834.9(17)**	**47.5(8)**
**C8a**	**-2274(3)**	**5131(3)**	**3113.2(17)**	**53.8(9)**
**C8b**	**1837(3)**	**7573(3)**	**3806.9(19)**	**56.9(10)**
**C9a**	**-2849(3)**	**5510(3)**	**2535.7(18)**	**58.9(10)**

C9b	1185(3)	8162(3)	3296(2)	62.8(11)
C10a	-2287(3)	5918(3)	2056.3(16)	52.1(9)
C10b	1749(3)	8587(3)	2825.0(19)	57.9(10)
C11a	438(3)	6048(2)	3782.8(15)	44.3(8)
C11b	5434(3)	6076(2)	3784.5(16)	46.5(8)
C12a	-573(3)	4704(3)	3843.8(16)	51.9(9)
C12b	3655(3)	6797(2)	4447.4(16)	51.1(9)
C13a	939(3)	6739(3)	772.5(16)	48.9(8)
C13b	2468(3)	10021(2)	1642.7(19)	56.6(10)
C14a	1590(4)	6288(3)	442.6(19)	58.2(10)
C14b	2092(3)	10012(2)	1073.4(19)	57.7(10)
C15a	2332(4)	6627(3)	40(2)	67.9(12)
C15b	1180(4)	10645(3)	733(2)	72.4(14)
C16a	2480(4)	7401(3)	-56(2)	66.0(11)
C16b	602(4)	11277(3)	918(3)	87.3(17)
C17a	1839(4)	7838(3)	280.0(19)	56.8(9)
C17b	979(5)	11275(3)	1461(3)	94.5(18)
C18a	1059(3)	7535(2)	690.9(17)	49.9(9)
C18b	1893(4)	10676(3)	1834(2)	76.2(13)
C19a	353(3)	7977(2)	1050.3(16)	47.5(8)
C19b	2369(5)	10662(3)	2414(3)	85.5(14)
C20a	299(3)	8753(3)	1053.9(19)	57.0(9)
C20b	1986(8)	11226(5)	2714(4)	135(3)
C21a	-429(4)	9086(3)	1416(2)	62.1(10)
C21b	2596(11)	11115(7)	3271(5)	188(4)
C22a	-1111(4)	8630(3)	1793(2)	60.5(10)
C22b	3487(9)	10450(6)	3509(4)	162(4)
C23a	-1029(3)	7850(3)	1779.8(16)	50.7(9)
C23b	3797(6)	9912(4)	3212(3)	99.0(18)
C24a	-1342(3)	6745(2)	678.0(16)	48.9(8)
C24b	4784(3)	9857(3)	1648.0(18)	58.2(10)
C25a	-1976(3)	7555(3)	349.0(16)	51.2(9)
C25b	5013(4)	10581(3)	1671(2)	66.2(11)
C26a	-2698(3)	7695(3)	-151.7(17)	60.4(11)
C26b	5702(5)	10951(3)	1243(3)	81.3(15)
C27a	-2842(4)	7089(3)	-357.4(19)	68.7(12)
C27b	6207(5)	10638(3)	798(3)	82.1(15)

**C28a**	**-2225(4)**	**6311(3)**	**-35(2)**	**68.3(12)**
**C28b**	**5956(4)**	**9925(3)**	**794(2)**	**69.2(12)**
**C29a**	**-1456(3)**	**6104(3)**	**475.7(17)**	**56.6(10)**
**C29b**	**5245(3)**	**9532(3)**	**1198.9(18)**	**55.1(9)**
**C30a**	**-739(4)**	**5285(3)**	**818.5(19)**	**61.3(10)**
**C30b**	**4947(3)**	**8784(3)**	**1203.5(18)**	**54.7(9)**
**C31a**	**-678(5)**	**4550(3)**	**718(3)**	**84.3(15)**
**C31b**	**5261(3)**	**8361(3)**	**811(2)**	**63.0(10)**
**C32a**	**62(6)**	**3827(4)**	**1072(3)**	**99.0(19)**
**C32b**	**4947(4)**	**7640(3)**	**889(2)**	**69.8(12)**
**C33a**	**742(6)**	**3835(3)**	**1538(3)**	**93.5(17)**
**C33b**	**4284(4)**	**7363(3)**	**1375(2)**	**66.8(11)**
**C34a**	**674(4)**	**4561(3)**	**1634(2)**	**69.1(12)**
**C34b**	**3958(3)**	**7811(2)**	**1747.2(19)**	**53.8(9)**

Table S3. Anisotropic Displacement Parameters ($Å^2 \times 10^3$) for Ir^{III}-p. The Anisotropic displacement factor exponent takes the form: $-2\pi^2[h^2a^{*2}U_{11}+2hka^*b^*U_{12}+\ldots]$.

**Atom**	**U_{11}**	**U_{22}**	**U_{33}**	**U_{12}**	**U_{13}**	**U_{23}**
**Ir1a**	**42.8(3)**	**58.2(3)**	**24.4(3)**	**-30.3(2)**	**1.30(17)**	**-2.09(19)**
**Ir1b**	**39.3(3)**	**50.7(3)**	**39.4(3)**	**-16.5(2)**	**-1.51(19)**	**12.0(2)**
**K1**	**24.3(8)**	**63.4(12)**	**21.4(9)**	**-18.7(8)**	**-7.7(7)**	**13.0(8)**
**F1a**	**112(2)**	**108(2)**	**99(2)**	**-65.2(19)**	**58.9(19)**	**-65(2)**
**F1b**	**93.4(18)**	**79.6(18)**	**75.7(19)**	**-33.8(15)**	**-40.1(16)**	**23.4(15)**
**F2a**	**78.8(15)**	**68.6(15)**	**79.9(18)**	**-47.0(13)**	**31.4(13)**	**-20.9(13)**
**F2b**	**144(3)**	**67(2)**	**147(4)**	**26(2)**	**-9(3)**	**-12(2)**
**F3a**	**67.7(13)**	**88.5(18)**	**42.1(13)**	**-22.4(13)**	**-9.4(11)**	**5.0(12)**
**F3b**	**157(3)**	**90(2)**	**113(3)**	**-86(2)**	**35(2)**	**-19(2)**
**F4a**	**121(2)**	**111(2)**	**75.3(19)**	**-56(2)**	**-15.7(17)**	**-39.4(18)**
**F4b**	**99(2)**	**100(2)**	**76.7(19)**	**-54.6(17)**	**43.3(16)**	**-18.2(17)**
**O1a**	**43.0(12)**	**86.3(19)**	**33.7(13)**	**-20.8(12)**	**-10.3(10)**	**-2.7(13)**
**O1b**	**50.5(13)**	**67.6(18)**	**50.2(16)**	**-22.3(13)**	**-2.6(12)**	**16.1(13)**
**O1w**	**60.5(15)**	**70.8(19)**	**56.4(17)**	**-20.4(14)**	**-0.7(13)**	**-2.7(14)**
**O2a**	**36.1(12)**	**84.1(19)**	**45.2(14)**	**-16.4(12)**	**-6.7(10)**	**-19.3(13)**
**O2b**	**56.4(14)**	**80.3(19)**	**39.5(14)**	**-28.6(14)**	**-5.7(11)**	**-0.7(13)**
**O2w**	**61.8(15)**	**81(2)**	**47.8(16)**	**-25.5(14)**	**-4.4(13)**	**-2.1(14)**
**O3a**	**50.3(14)**	**66.6(17)**	**45.7(15)**	**-18.0(12)**	**-4.8(11)**	**6.5(13)**

O3b	36.9(11)	57.4(15)	60.7(16)	-14.5(10)	-12.2(11)	-4.8(13)
O4a	54.6(14)	95(2)	34.0(14)	-31.8(14)	0.2(11)	2.3(14)
O4b	43.2(12)	63.3(16)	47.9(15)	-16.3(11)	-13.7(11)	10.6(12)
N1a	40.1(13)	62.5(18)	36.8(15)	-22.7(13)	0.3(11)	-6.7(13)
N1b	41.6(14)	52.9(17)	40.6(16)	-16.4(12)	-5.0(12)	4.4(13)
N2a	44.6(14)	58.9(17)	28.9(14)	-28.3(13)	-1.1(11)	-0.9(12)
N2b	40.2(14)	54.6(18)	39.1(16)	-13.9(12)	-2.0(12)	9.7(13)
N3a	46.7(14)	55.4(17)	26.5(13)	-25.1(12)	-1.1(11)	-2.5(12)
N3b	74(2)	72(2)	50.0(19)	-18.9(16)	3.7(15)	-2.0(15)
N4a	63.0(18)	52.9(18)	45.0(17)	-30.7(15)	3.1(14)	-3.3(14)
N4b	41.7(14)	52.7(17)	37.5(15)	-18.1(12)	0.7(11)	2.4(13)
C1a	44.3(17)	71(2)	37.4(19)	-26.8(17)	1.9(14)	-7.2(17)
C1b	42.1(17)	70(3)	51(2)	-25.8(17)	-4.1(16)	6.9(19)
C2a	36.7(16)	78(3)	43(2)	-26.1(16)	0.8(14)	-7.3(18)
C2b	43.4(18)	74(3)	53(2)	-29.7(18)	-1.5(16)	1(2)
C3a	36.6(15)	68(2)	37.2(18)	-20.5(15)	-6.8(13)	-2.0(16)
C3b	34.4(15)	63(2)	40.1(19)	-17.8(15)	-7.8(13)	5.6(16)
C4a	34.9(15)	57(2)	33.9(17)	-18.2(14)	-5.2(13)	1.4(15)
C4b	37.1(15)	63(2)	30.7(16)	-20.8(15)	-7.3(13)	3.2(15)
C5a	35.9(14)	51.4(18)	27.6(15)	-16.4(13)	-3.1(12)	-0.7(14)
C5b	35.8(15)	52.4(19)	31.6(16)	-15.7(14)	-5.0(12)	5.3(14)
C6a	39.1(15)	54.5(19)	30.3(16)	-22.1(14)	-1.3(13)	0.0(14)
C6b	37.0(15)	51.0(19)	37.9(18)	-15.2(14)	-2.8(13)	5.3(15)
C7a	43.1(16)	61(2)	32.5(17)	-25.5(15)	1.0(13)	-5.0(15)
C7b	40.4(16)	47.4(19)	42.0(19)	-14.7(14)	0.1(14)	2.4(15)
C8a	52.4(19)	72(2)	38.0(19)	-37.3(18)	4.4(15)	-6.2(17)
C8b	42.5(17)	58(2)	50(2)	-12.5(16)	5.8(15)	6.6(18)
C9a	51.0(19)	91(3)	40(2)	-42(2)	-2.3(16)	-12.3(19)
C9b	37.6(17)	64(2)	60(2)	-6.1(16)	2.2(16)	7(2)
C10a	48.9(18)	77(3)	31.4(17)	-39.7(18)	-3.9(14)	-2.7(17)
C10b	38.8(17)	58(2)	51(2)	-6.3(15)	-1.1(15)	8.7(18)
C11a	35.8(15)	61(2)	30.6(16)	-19.7(14)	-7.1(13)	-3.3(15)
C11b	32.5(15)	57(2)	36.9(18)	-12.7(14)	-5.0(13)	1.3(15)
C12a	49.0(19)	68(2)	32.6(18)	-31.1(17)	0.7(14)	1.9(16)
C12b	45.0(17)	59(2)	34.3(18)	-13.8(15)	4.7(14)	2.8(16)
C13a	56.3(19)	67(2)	28.8(16)	-34.8(18)	5.3(14)	-10.6(16)
C13b	49.8(19)	46(2)	52(2)	-15.9(16)	-4.3(17)	12.9(17)

**C14a**	**71(2)**	**71(3)**	**49(2)**	**-43(2)**	**17.3(18)**	**-25(2)**
**C14b**	**51.0(19)**	**53(2)**	**49(2)**	**-20.7(16)**	**-7.4(16)**	**13.1(17)**
**C15a**	**77(3)**	**87(3)**	**63(3)**	**-45(2)**	**29(2)**	**-39(2)**
**C15b**	**63(2)**	**60(3)**	**66(3)**	**-29(2)**	**-16(2)**	**22(2)**
**C16a**	**70(2)**	**86(3)**	**57(2)**	**-47(2)**	**24(2)**	**-26(2)**
**C16b**	**70(3)**	**62(3)**	**85(4)**	**-7(2)**	**-18(3)**	**23(3)**
**C17a**	**67(2)**	**63(2)**	**47(2)**	**-37.6(19)**	**11.9(18)**	**-14.0(19)**
**C17b**	**93(4)**	**51(3)**	**102(5)**	**0(2)**	**-4(3)**	**8(3)**
**C18a**	**50.7(18)**	**60(2)**	**39.8(19)**	**-30.9(16)**	**0.2(15)**	**-6.4(16)**
**C18b**	**76(3)**	**54(3)**	**73(3)**	**-14(2)**	**4(2)**	**8(2)**
**C19a**	**52.2(18)**	**56(2)**	**35.5(17)**	**-28.9(16)**	**0.8(14)**	**-6.5(15)**
**C19b**	**104(3)**	**68(3)**	**68(3)**	**-12(2)**	**3(2)**	**-9.9(19)**
**C20a**	**63(2)**	**64(2)**	**49(2)**	**-34.4(19)**	**6.7(18)**	**-14.0(19)**
**C20b**	**168(6)**	**102(4)**	**117(5)**	**15(3)**	**-9(4)**	**-49(3)**
**C21a**	**66(2)**	**61(2)**	**65(3)**	**-29.3(19)**	**9(2)**	**-19(2)**
**C21b**	**248(9)**	**154(6)**	**143(6)**	**40(5)**	**-51(4)**	**-89(4)**
**C22a**	**61(2)**	**65(3)**	**57(2)**	**-25.0(19)**	**10.2(18)**	**-17(2)**
**C22b**	**208(8)**	**148(6)**	**117(5)**	**20(4)**	**-39(4)**	**-75(3)**
**C23a**	**58.1(19)**	**66(2)**	**32.5(17)**	**-32.8(18)**	**6.7(15)**	**-10.7(16)**
**C23b**	**125(4)**	**98(4)**	**63(3)**	**-9(3)**	**-13(2)**	**-26(2)**
**C24a**	**53.3(18)**	**64(2)**	**30.8(17)**	**-32.8(17)**	**3.0(14)**	**-6.3(16)**
**C24b**	**57(2)**	**56(2)**	**45(2)**	**-25.2(17)**	**-5.4(17)**	**12.0(18)**
**C25a**	**53.4(18)**	**65(2)**	**30.1(17)**	**-26.9(17)**	**0.7(14)**	**-2.6(16)**
**C25b**	**78(3)**	**60(2)**	**53(2)**	**-33(2)**	**3(2)**	**0(2)**
**C26a**	**58(2)**	**81(3)**	**32.5(19)**	**-28(2)**	**-2.9(16)**	**1.2(19)**
**C26b**	**100(3)**	**67(3)**	**75(3)**	**-50(3)**	**13(3)**	**-3(3)**
**C27a**	**73(3)**	**97(3)**	**39(2)**	**-41(3)**	**-8.6(19)**	**-13(2)**
**C27b**	**86(3)**	**82(3)**	**71(3)**	**-52(3)**	**14(3)**	**4(3)**
**C28a**	**88(3)**	**90(3)**	**45(2)**	**-54(3)**	**4(2)**	**-25(2)**
**C28b**	**69(2)**	**72(3)**	**57(3)**	**-33(2)**	**9(2)**	**-2(2)**
**C29a**	**70(2)**	**75(3)**	**36.1(19)**	**-46(2)**	**7.6(17)**	**-15.2(18)**
**C29b**	**51.2(19)**	**60(2)**	**43(2)**	**-22.1(17)**	**-0.6(16)**	**2.5(17)**
**C30a**	**78(3)**	**69(3)**	**47(2)**	**-44(2)**	**7.5(19)**	**-15.2(19)**
**C30b**	**43.2(17)**	**65(2)**	**44(2)**	**-18.9(16)**	**1.8(15)**	**-0.5(18)**
**C31a**	**120(4)**	**78(3)**	**71(3)**	**-52(3)**	**3(3)**	**-28(3)**
**C31b**	**56(2)**	**75(3)**	**51(2)**	**-24(2)**	**8.5(18)**	**-10(2)**
**C32a**	**149(5)**	**66(3)**	**95(4)**	**-48(4)**	**2(4)**	**-29(3)**

**C32b**	**67(2)**	**82(3)**	**60(3)**	**-29(2)**	**12(2)**	**-18(2)**
**C33a**	**127(5)**	**59(3)**	**85(4)**	**-27(3)**	**0(3)**	**-11(3)**
**C33b**	**65(2)**	**64(3)**	**68(3)**	**-27(2)**	**2(2)**	**-11(2)**
**C34a**	**82(3)**	**59(3)**	**63(3)**	**-31(2)**	**3(2)**	**-8(2)**
**C34b**	**49.9(18)**	**52(2)**	**53(2)**	**-20.1(16)**	**2.8(16)**	**-6.3(18)**

Table S4. Bond Lengths for Ir^{III}-p.

Atom	Atom	Length/Å		Atom	Atom	Length/Å
**Ir1a**	**N1a**	**2.125(3)**		**C4a**	**C5a**	**1.392(5)**
**Ir1a**	**N2a**	**2.133(3)**		**C4a**	**C11a**	**1.509(5)**
**Ir1a**	**N3a**	**2.040(3)**		**C4b**	**C5b**	**1.402(5)**
**Ir1a**	**N4a**	**2.040(3)**		**C4b**	**C11b**	**1.501(5)**
**Ir1a**	**C13a**	**2.014(3)**		**C5a**	**C6a**	**1.505(4)**
**Ir1a**	**C24a**	**2.005(3)**		**C5b**	**C6b**	**1.495(4)**
**Ir1b**	**N1b**	**2.120(3)**		**C6a**	**C7a**	**1.394(5)**
**Ir1b**	**N2b**	**2.135(3)**		**C6b**	**C7b**	**1.409(5)**
**Ir1b**	**N3b**	**2.018(4)**		**C7a**	**C8a**	**1.389(5)**
**Ir1b**	**N4b**	**2.051(3)**		**C7a**	**C12a**	**1.520(5)**
**Ir1b**	**C13b**	**1.995(3)**		**C7b**	**C8b**	**1.391(5)**
**Ir1b**	**C24b**	**2.014(4)**		**C7b**	**C12b**	**1.522(5)**
**K1**	**O1a**	**2.266(3)**		**C8a**	**C9a**	**1.383(5)**
**K1**	**O1b**	**2.291(3)**		**C8b**	**C9b**	**1.378(5)**
**K1**	**O1w**	**2.393(4)**		**C9a**	**C10a**	**1.374(5)**
**K1**	**O2w**	**2.393(3)**		**C9b**	**C10b**	**1.377(5)**
**K1**	**O4a**[1]	**2.300(3)**		**C13a**	**C14a**	**1.393(6)**
**K1**	**O4b**[2]	**2.270(3)**		**C13a**	**C18a**	**1.414(5)**
**K1**	**C11a**	**3.473(3)**		**C13b**	**C14b**	**1.412(6)**
**K1**	**C11b**[2]	**3.470(3)**		**C13b**	**C18b**	**1.412(7)**
**K1**	**C12a**[1]	**3.491(4)**		**C14a**	**C15a**	**1.374(6)**
**K1**	**C12b**	**3.487(4)**		**C14b**	**C15b**	**1.382(6)**
**F1a**	**C15a**	**1.346(5)**		**C15a**	**C16a**	**1.381(6)**
**F1b**	**C15b**	**1.356(6)**		**C15b**	**C16b**	**1.371(9)**
**F2a**	**C17a**	**1.348(5)**		**C16a**	**C17a**	**1.383(7)**
**F2b**	**C17b**	**1.363(8)**		**C16b**	**C17b**	**1.348(9)**
**F3a**	**C26a**	**1.355(5)**		**C17a**	**C18a**	**1.396(5)**
**F3b**	**C26b**	**1.356(6)**		**C17b**	**C18b**	**1.385(7)**
**F4a**	**C28a**	**1.370(5)**		**C18a**	**C19a**	**1.462(6)**

**F4b**	**C28b**	**1.355(6)**		**C18b**	**C19b**	**1.464(8)**
**O1a**	**C11a**	**1.249(4)**		**C19a**	**C20a**	**1.376(6)**
**O1b**	**C12b**	**1.245(4)**		**C19b**	**C20b**	**1.399(9)**
**O2a**	**C11a**	**1.243(4)**		**C20a**	**C21a**	**1.375(6)**
**O2b**	**C12b**	**1.253(4)**		**C20b**	**C21b**	**1.427(12)**
**O3a**	**C12a**	**1.243(5)**		**C21a**	**C22a**	**1.386(6)**
**O3b**	**C11b**	**1.248(4)**		**C21b**	**C22b**	**1.344(13)**
**O4a**	**C12a**	**1.252(5)**		**C22a**	**C23a**	**1.385(6)**
**O4b**	**C11b**	**1.240(4)**		**C22b**	**C23b**	**1.347(10)**
**N1a**	**C1a**	**1.347(4)**		**C24a**	**C25a**	**1.405(6)**
**N1a**	**C5a**	**1.361(4)**		**C24a**	**C29a**	**1.417(6)**
**N1b**	**C1b**	**1.354(4)**		**C24b**	**C25b**	**1.408(6)**
**N1b**	**C5b**	**1.353(4)**		**C24b**	**C29b**	**1.403(7)**
**N2a**	**C6a**	**1.357(4)**		**C25a**	**C26a**	**1.378(5)**
**N2a**	**C10a**	**1.351(4)**		**C25b**	**C26b**	**1.379(6)**
**N2b**	**C6b**	**1.353(4)**		**C26a**	**C27a**	**1.372(7)**
**N2b**	**C10b**	**1.343(5)**		**C26b**	**C27b**	**1.399(8)**
**N3a**	**C19a**	**1.377(4)**		**C27a**	**C28a**	**1.357(7)**
**N3a**	**C23a**	**1.344(5)**		**C27b**	**C28b**	**1.384(7)**
**N3b**	**C19b**	**1.367(7)**		**C28a**	**C29a**	**1.403(6)**
**N3b**	**C23b**	**1.342(7)**		**C28b**	**C29b**	**1.388(6)**
**N4a**	**C30a**	**1.382(5)**		**C29a**	**C30a**	**1.457(6)**
**N4a**	**C34a**	**1.332(6)**		**C29b**	**C30b**	**1.466(6)**
**N4b**	**C30b**	**1.374(5)**		**C30a**	**C31a**	**1.395(7)**
**N4b**	**C34b**	**1.347(5)**		**C30b**	**C31b**	**1.366(6)**
**C1a**	**C2a**	**1.377(5)**		**C31a**	**C32a**	**1.366(9)**
**C1b**	**C2b**	**1.357(6)**		**C31b**	**C32b**	**1.384(7)**
**C2a**	**C3a**	**1.372(5)**		**C32a**	**C33a**	**1.374(8)**
**C2b**	**C3b**	**1.385(5)**		**C32b**	**C33b**	**1.391(6)**
**C3a**	**C4a**	**1.402(4)**		**C33a**	**C34a**	**1.374(7)**
**C3b**	**C4b**	**1.396(4)**		**C33b**	**C34b**	**1.360(7)**

Table S5. Bond Angles for Ir^{III}-p.

Atom	Atom	Atom	Angle/°		Atom	Atom	Atom	Angle/°
**N2a**	**Ir1a**	**N1a**	**75.92(10)**		**C5b**	**C6b**	**N2b**	**113.8(3)**
**N3a**	**Ir1a**	**N1a**	**85.51(11)**		**C7b**	**C6b**	**N2b**	**120.0(3)**
**N3a**	**Ir1a**	**N2a**	**96.47(12)**		**C7b**	**C6b**	**C5b**	**126.1(3)**

N4a	Ir1a	N1a	98.53(13)		C8a	C7a	C6a	118.4(3)
N4a	Ir1a	N2a	87.47(12)		C12a	C7a	C6a	125.2(3)
N4a	Ir1a	N3a	174.96(11)		C12a	C7a	C8a	116.3(3)
C13a	Ir1a	N1a	96.88(12)		C8b	C7b	C6b	117.9(3)
C13a	Ir1a	N2a	172.48(11)		C12b	C7b	C6b	125.9(3)
C13a	Ir1a	N3a	80.61(14)		C12b	C7b	C8b	116.1(3)
C13a	Ir1a	N4a	95.85(15)		C9a	C8a	C7a	120.2(3)
C24a	Ir1a	N1a	176.67(12)		C9b	C8b	C7b	120.9(3)
C24a	Ir1a	N2a	100.76(12)		C10a	C9a	C8a	118.7(3)
C24a	Ir1a	N3a	95.21(14)		C10b	C9b	C8b	118.1(3)
C24a	Ir1a	N4a	80.93(15)		C9a	C10a	N2a	121.7(3)
C24a	Ir1a	C13a	86.45(14)		C9b	C10b	N2b	122.2(3)
N2b	Ir1b	N1b	75.96(10)		O1a	C11a	K1	12.13(19)
N3b	Ir1b	N1b	98.15(14)		O2a	C11a	K1	133.7(2)
N3b	Ir1b	N2b	87.43(15)		O2a	C11a	O1a	124.4(3)
N4b	Ir1b	N1b	86.03(12)		C4a	C11a	K1	108.06(19)
N4b	Ir1b	N2b	96.23(13)		C4a	C11a	O1a	117.5(3)
N4b	Ir1b	N3b	175.03(13)		C4a	C11a	O2a	118.0(3)
C13b	Ir1b	N1b	176.01(13)		O3b	C11b	K1[2]	133.6(2)
C13b	Ir1b	N2b	100.18(12)		O4b	C11b	K1[2]	11.75(19)
C13b	Ir1b	N3b	80.53(17)		O4b	C11b	O3b	124.3(3)
C13b	Ir1b	N4b	95.48(16)		C4b	C11b	K1[2]	108.36(18)
C24b	Ir1b	N1b	98.19(13)		C4b	C11b	O3b	117.9(3)
C24b	Ir1b	N2b	173.35(14)		C4b	C11b	O4b	117.8(3)
C24b	Ir1b	N3b	96.63(18)		O3a	C12a	K1[1]	129.4(2)
C24b	Ir1b	N4b	80.04(16)		O4a	C12a	K1[1]	14.4(2)
C24b	Ir1b	C13b	85.72(15)		O4a	C12a	O3a	124.1(3)
O1b	K1	O1a	87.77(11)		C7a	C12a	K1[1]	110.2(2)
O1w	K1	O1a	101.41(12)		C7a	C12a	O3a	119.6(3)
O1w	K1	O1b	85.85(12)		C7a	C12a	O4a	116.1(3)
O2w	K1	O1a	81.31(11)		O1b	C12b	K1	13.1(2)
O2w	K1	O1b	168.01(12)		O2b	C12b	K1	129.5(2)
O2w	K1	O1w	91.44(13)		O2b	C12b	O1b	124.4(3)
O4a[1]	K1	O1a	89.14(11)		C7b	C12b	K1	110.8(2)
O4a[1]	K1	O1b	98.84(14)		C7b	C12b	O1b	116.6(3)
O4a[1]	K1	O1w	168.66(12)		C7b	C12b	O2b	118.9(3)
O4a[1]	K1	O2w	86.00(12)		C14a	C13a	Ir1a	127.0(3)

O4b[2]	K1	O1a	175.70(15)		C18a	C13a	Ir1a	113.5(3)
O4b[2]	K1	O1b	89.98(10)		C18a	C13a	C14a	119.5(3)
O4b[2]	K1	O1w	82.08(11)		C14b	C13b	Ir1b	126.9(3)
O4b[2]	K1	O2w	101.22(11)		C18b	C13b	Ir1b	114.7(3)
O4b[2]	K1	O4a[1]	87.58(11)		C18b	C13b	C14b	118.3(4)
C11a	K1	O1a	6.65(10)		C15a	C14a	C13a	119.4(4)
C11a	K1	O1b	91.04(9)		C15b	C14b	C13b	118.2(5)
C11a	K1	O1w	95.88(10)		C14a	C15a	F1a	119.0(4)
C11a	K1	O2w	77.60(10)		C16a	C15a	F1a	117.7(4)
C11a	K1	O4a[1]	94.36(10)		C16a	C15a	C14a	123.3(4)
C11a	K1	O4b[2]	177.64(13)		C14b	C15b	F1b	117.7(5)
C11b[2]	K1	O1a	177.05(11)		C16b	C15b	F1b	118.2(4)
C11b[2]	K1	O1b	95.17(10)		C16b	C15b	C14b	124.1(5)
C11b[2]	K1	O1w	78.79(10)		C17a	C16a	C15a	116.6(4)
C11b[2]	K1	O2w	95.75(10)		C17b	C16b	C15b	116.6(4)
C11b[2]	K1	O4a[1]	90.47(10)		C16a	C17a	F2a	116.7(3)
C11b[2]	K1	O4b[2]	6.39(11)		C18a	C17a	F2a	120.2(4)
C11b[2]	K1	C11a	171.47(11)		C18a	C17a	C16a	123.0(4)
C12a[1]	K1	O1a	92.03(10)		C16b	C17b	F2b	116.4(5)
C12a[1]	K1	O1b	106.15(12)		C18b	C17b	F2b	119.7(6)
C12a[1]	K1	O1w	162.42(12)		C18b	C17b	C16b	123.8(6)
C12a[1]	K1	O2w	79.32(11)		C17a	C18a	C13a	118.1(4)
C12a[1]	K1	O4a[1]	7.77(10)		C19a	C18a	C13a	116.9(3)
C12a[1]	K1	O4b[2]	85.07(10)		C19a	C18a	C17a	125.0(4)
C12a[1]	K1	C11a	96.68(9)		C17b	C18b	C13b	118.9(6)
C12a[1]	K1	C11b[2]	87.22(9)		C19b	C18b	C13b	114.9(4)
C12b	K1	O1a	85.39(10)		C19b	C18b	C17b	126.1(6)
C12b	K1	O1b	7.06(11)		C18a	C19a	N3a	112.2(3)
C12b	K1	O1w	79.84(11)		C20a	C19a	N3a	119.3(4)
C12b	K1	O2w	162.35(11)		C20a	C19a	C18a	128.5(3)
C12b	K1	O4a[1]	105.42(12)		C18b	C19b	N3b	113.0(5)
C12b	K1	O4b[2]	92.80(10)		C20b	C19b	N3b	119.2(6)
C12b	K1	C11a	87.98(9)		C20b	C19b	C18b	127.7(6)
C12b	K1	C11b[2]	97.53(9)		C21a	C20a	C19a	121.3(4)
C12b	K1	C12a[1]	112.84(11)		C21b	C20b	C19b	119.0(7)
C11a	O1a	K1	161.2(3)		C22a	C21a	C20a	119.5(4)
C12b	O1b	K1	159.9(3)		C22b	C21b	C20b	119.2(9)

C12a	O4a	K1[1]	157.8(3)		C23a	C22a	C21a	117.5(4)
C11b	O4b	K1[2]	161.9(3)		C23b	C22b	C21b	119.3(8)
C1a	N1a	Ir1a	122.1(2)		C22a	C23a	N3a	123.2(3)
C5a	N1a	Ir1a	115.4(2)		C22b	C23b	N3b	124.2(7)
C5a	N1a	C1a	119.7(3)		C25a	C24a	Ir1a	127.3(3)
C1b	N1b	Ir1b	122.4(2)		C29a	C24a	Ir1a	114.0(3)
C5b	N1b	Ir1b	116.1(2)		C29a	C24a	C25a	118.6(3)
C5b	N1b	C1b	119.4(3)		C25b	C24b	Ir1b	125.3(4)
C6a	N2a	Ir1a	116.9(2)		C29b	C24b	Ir1b	114.4(3)
C10a	N2a	Ir1a	122.3(2)		C29b	C24b	C25b	120.3(4)
C10a	N2a	C6a	119.8(3)		C26a	C25a	C24a	119.0(4)
C6b	N2b	Ir1b	116.2(2)		C26b	C25b	C24b	118.0(5)
C10b	N2b	Ir1b	122.6(2)		C25a	C26a	F3a	117.9(4)
C10b	N2b	C6b	120.3(3)		C27a	C26a	F3a	118.0(3)
C19a	N3a	Ir1a	116.7(3)		C27a	C26a	C25a	124.1(4)
C23a	N3a	Ir1a	124.1(2)		C25b	C26b	F3b	119.1(5)
C23a	N3a	C19a	119.2(3)		C27b	C26b	F3b	117.1(4)
C19b	N3b	Ir1b	116.8(4)		C27b	C26b	C25b	123.8(5)
C23b	N3b	Ir1b	124.3(4)		C28a	C27a	C26a	116.2(4)
C23b	N3b	C19b	118.9(5)		C28b	C27b	C26b	116.2(4)
C30a	N4a	Ir1a	115.8(3)		C27a	C28a	F4a	116.6(4)
C34a	N4a	Ir1a	124.8(3)		C29a	C28a	F4a	119.2(5)
C34a	N4a	C30a	119.5(4)		C29a	C28a	C27a	124.2(4)
C30b	N4b	Ir1b	116.7(3)		C27b	C28b	F4b	116.2(4)
C34b	N4b	Ir1b	124.2(3)		C29b	C28b	F4b	120.6(4)
C34b	N4b	C30b	119.1(4)		C29b	C28b	C27b	123.2(5)
C2a	C1a	N1a	122.0(3)		C28a	C29a	C24a	117.8(4)
C2b	C1b	N1b	122.6(3)		C30a	C29a	C24a	116.1(3)
C3a	C2a	C1a	118.6(3)		C30a	C29a	C28a	126.1(4)
C3b	C2b	C1b	118.2(3)		C28b	C29b	C24b	118.6(4)
C4a	C3a	C2a	120.5(3)		C30b	C29b	C24b	116.6(3)
C4b	C3b	C2b	120.6(3)		C30b	C29b	C28b	124.8(4)
C5a	C4a	C3a	117.9(3)		C29a	C30a	N4a	113.3(4)
C11a	C4a	C3a	116.6(3)		C31a	C30a	N4a	118.7(4)
C11a	C4a	C5a	125.2(3)		C31a	C30a	C29a	128.0(4)
C5b	C4b	C3b	117.5(3)		C29b	C30b	N4b	112.3(4)
C11b	C4b	C3b	116.8(3)		C31b	C30b	N4b	119.7(4)

C11b	C4b	C5b	125.4(3)		C31b	C30b	C29b	128.0(4)
C4a	C5a	N1a	120.6(3)		C32a	C31a	C30a	121.3(5)
C6a	C5a	N1a	112.8(3)		C32b	C31b	C30b	121.3(4)
C6a	C5a	C4a	126.6(3)		C33a	C32a	C31a	118.3(5)
C4b	C5b	N1b	120.6(3)		C33b	C32b	C31b	118.0(5)
C6b	C5b	N1b	112.8(3)		C34a	C33a	C32a	119.9(5)
C6b	C5b	C4b	126.6(3)		C34b	C33b	C32b	119.2(4)
C5a	C6a	N2a	112.6(3)		C33a	C34a	N4a	122.2(5)
C7a	C6a	N2a	120.6(3)		C33b	C34b	N4b	122.6(4)
C7a	C6a	C5a	126.6(3)					

Table S6. Torsion Angles for Ir^{III}-p.

A	B	C	D	Angle/˚		A	B	C	D	Angle/˚
Ir1a	N1a	C1a	C2a	158.3(3)		N3b	C19b	C18b	C13b	-1.5(5)
Ir1a	N1a	C5a	C4a	-152.6(2)		N3b	C19b	C18b	C17b	-179.1(5)
Ir1a	N1a	C5a	C6a	26.8(3)		N3b	C19b	C20b	C21b	3.2(9)
Ir1a	N2a	C6a	C5a	14.7(3)		N3b	C23b	C22b	C21b	1.1(10)
Ir1a	N2a	C6a	C7a	-160.9(3)		N4a	C30a	C29a	C24a	-0.8(4)
Ir1a	N2a	C10a	C9a	165.9(3)		N4a	C30a	C29a	C28a	178.3(3)
Ir1a	N3a	C19a	C18a	1.7(2)		N4a	C30a	C31a	C32a	-0.6(5)
Ir1a	N3a	C19a	C20a	-179.0(3)		N4a	C34a	C33a	C32a	0.4(6)
Ir1a	N3a	C23a	C22a	179.3(3)		N4b	C30b	C29b	C24b	-1.5(4)
Ir1a	N4a	C30a	C29a	0.3(3)		N4b	C30b	C29b	C28b	177.3(3)
Ir1a	N4a	C30a	C31a	179.5(3)		N4b	C30b	C31b	C32b	-2.6(4)
Ir1a	N4a	C34a	C33a	-179.3(4)		N4b	C34b	C33b	C32b	-1.1(4)
Ir1a	C13a	C14a	C15a	-179.1(4)		C1a	N1a	C5a	C4a	8.9(4)
Ir1a	C13a	C18a	C17a	178.5(3)		C1a	N1a	C5a	C6a	-171.7(4)
Ir1a	C13a	C18a	C19a	-1.2(3)		C1a	C2a	C3a	C4a	4.9(5)
Ir1a	C24a	C25a	C26a	177.0(3)		C1b	N1b	C5b	C4b	-10.1(4)
Ir1a	C24a	C29a	C28a	-178.3(3)		C1b	N1b	C5b	C6b	171.8(4)
Ir1a	C24a	C29a	C30a	0.9(3)		C1b	C2b	C3b	C4b	-4.7(5)
Ir1b	N1b	C1b	C2b	-161.2(4)		C2a	C1a	N1a	C5a	-1.9(5)
Ir1b	N1b	C5b	C4b	154.0(3)		C2a	C3a	C4a	C5a	1.8(5)
Ir1b	N1b	C5b	C6b	-24.0(3)		C2a	C3a	C4a	C11a	-172.1(4)
Ir1b	N2b	C6b	C5b	-14.3(3)		C2b	C1b	N1b	C5b	1.9(6)
Ir1b	N2b	C6b	C7b	162.0(3)		C2b	C3b	C4b	C5b	-3.0(5)
Ir1b	N2b	C10b	C9b	-166.9(4)		C2b	C3b	C4b	C11b	171.0(4)

Ir1b	N3b	C19b	C18b	1.5(3)		C3a	C4a	C5a	C6a	171.9(3)
Ir1b	N3b	C19b	C20b	-179.6(6)		C3b	C4b	C5b	C6b	-171.7(3)
Ir1b	N3b	C23b	C22b	177.2(8)		C4a	C5a	C6a	C7a	-32.3(5)
Ir1b	N4b	C30b	C29b	2.5(2)		C4b	C5b	C6b	C7b	31.1(5)
Ir1b	N4b	C30b	C31b	-177.5(3)		C5a	C6a	N2a	C10a	-176.6(3)
Ir1b	N4b	C34b	C33b	179.3(3)		C5a	C6a	C7a	C8a	177.2(4)
Ir1b	C13b	C14b	C15b	-177.6(3)		C5a	C6a	C7a	C12a	-5.9(5)
Ir1b	C13b	C18b	C17b	178.6(4)		C5b	C6b	N2b	C10b	176.4(4)
Ir1b	C13b	C18b	C19b	0.8(4)		C5b	C6b	C7b	C8b	-176.4(4)
Ir1b	C24b	C25b	C26b	-179.1(4)		C5b	C6b	C7b	C12b	7.6(5)
Ir1b	C24b	C29b	C28b	-179.1(3)		C6a	N2a	C10a	C9a	-2.2(5)
Ir1b	C24b	C29b	C30b	-0.2(3)		C6a	C5a	C4a	C11a	-14.8(4)
K1	O1a	C11a	O2a	143.0(7)		C6a	C7a	C8a	C9a	2.2(5)
K1	O1a	C11a	C4a	-40.7(7)		C6b	N2b	C10b	C9b	1.6(5)
K1	O1b	C12b	O2b	-117.2(7)		C6b	C5b	C4b	C11b	14.8(5)
K1	O1b	C12b	C7b	66.0(7)		C6b	C7b	C8b	C9b	-2.9(5)
K1[1]	O4a	C12a	O3a	116.4(6)		C7a	C6a	N2a	C10a	7.8(5)
K1[1]	O4a	C12a	C7a	-68.6(6)		C7a	C8a	C9a	C10a	3.2(5)
K1[2]	O4b	C11b	O3b	-145.3(8)		C7b	C6b	N2b	C10b	-7.3(5)
K1[2]	O4b	C11b	C4b	38.1(8)		C7b	C8b	C9b	C10b	-2.5(5)
K1	C11a	C4a	C3a	-38.2(3)		C9a	C8a	C7a	C12a	-174.9(4)
K1	C11a	C4a	C5a	148.4(2)		C9b	C8b	C7b	C12b	173.6(4)
K1[2]	C11b	C4b	C3b	38.8(3)		C13a	C14a	C15a	C16a	0.6(5)
K1[2]	C11b	C4b	C5b	-147.7(2)		C13a	C18a	C17a	C16a	1.1(4)
K1[1]	C12a	C7a	C6a	138.8(3)		C13a	C18a	C19a	C20a	-179.5(3)
K1[1]	C12a	C7a	C8a	-44.2(3)		C13b	C14b	C15b	C16b	-1.1(5)
K1	C12b	C7b	C6b	-138.7(3)		C13b	C18b	C17b	C16b	-0.4(5)
K1	C12b	C7b	C8b	45.2(3)		C13b	C18b	C19b	C20b	179.8(6)
F1a	C15a	C14a	C13a	-179.4(4)		C14a	C13a	C18a	C17a	-0.3(4)
F1a	C15a	C16a	C17a	-179.8(4)		C14a	C13a	C18a	C19a	-180.0(3)
F1b	C15b	C14b	C13b	-179.6(3)		C14a	C15a	C16a	C17a	0.2(6)
F1b	C15b	C16b	C17b	179.9(5)		C14b	C13b	C18b	C17b	0.7(5)
F2a	C17a	C16a	C15a	178.6(4)		C14b	C13b	C18b	C19b	-177.1(4)
F2a	C17a	C18a	C13a	-178.5(3)		C14b	C15b	C16b	C17b	1.4(5)
F2a	C17a	C18a	C19a	1.1(5)		C15a	C14a	C13a	C18a	-0.5(5)
F2b	C17b	C16b	C15b	179.9(5)		C15a	C16a	C17a	C18a	-1.1(5)
F2b	C17b	C18b	C13b	179.1(5)		C15b	C14b	C13b	C18b	-0.0(4)

F2b	C17b	C18b	C19b	-3.3(7)		C15b	C16b	C17b	C18b	-0.6(6)
F3a	C26a	C25a	C24a	179.8(3)		C16a	C17a	C18a	C19a	-179.2(4)
F3a	C26a	C27a	C28a	-179.7(4)		C16b	C17b	C18b	C19b	177.2(6)
F3b	C26b	C25b	C24b	178.3(4)		C17a	C18a	C19a	C20a	0.8(5)
F3b	C26b	C27b	C28b	-178.3(5)		C17b	C18b	C19b	C20b	2.1(8)
F4a	C28a	C27a	C26a	-179.6(4)		C18a	C19a	N3a	C23a	179.4(3)
F4a	C28a	C29a	C24a	-179.7(4)		C18a	C19a	C20a	C21a	179.1(4)
F4a	C28a	C29a	C30a	1.3(5)		C18b	C19b	N3b	C23b	-179.9(5)
F4b	C28b	C27b	C26b	179.0(5)		C18b	C19b	C20b	C21b	-178.1(10)
F4b	C28b	C29b	C24b	179.3(4)		C19a	N3a	C23a	C22a	1.8(4)
F4b	C28b	C29b	C30b	0.5(5)		C19a	C20a	C21a	C22a	0.9(5)
O1a	C11a	C4a	C3a	-29.9(4)		C19b	N3b	C23b	C22b	-1.2(8)
O1a	C11a	C4a	C5a	156.7(3)		C19b	C20b	C21b	C22b	-3.3(11)
O1b	C12b	C7b	C6b	-151.5(4)		C20a	C19a	N3a	C23a	-1.3(4)
O1b	C12b	C7b	C8b	32.4(5)		C20a	C21a	C22a	C23a	-0.5(5)
O2a	C11a	C4a	C3a	146.6(3)		C20b	C19b	N3b	C23b	-1.1(8)
O2a	C11a	C4a	C5a	-26.7(4)		C20b	C21b	C22b	C23b	1.2(15)
O2b	C12b	C7b	C6b	31.6(5)		C24a	C25a	C26a	C27a	-0.2(4)
O2b	C12b	C7b	C8b	-144.5(4)		C24a	C29a	C28a	C27a	1.9(5)
O3a	C12a	C7a	C6a	-31.7(5)		C24a	C29a	C30a	C31a	-179.8(4)
O3a	C12a	C7a	C8a	145.2(4)		C24b	C25b	C26b	C27b	-1.5(5)
O3b	C11b	C4b	C3b	-145.7(4)		C24b	C29b	C28b	C27b	-2.0(5)
O3b	C11b	C4b	C5b	27.8(4)		C24b	C29b	C30b	C31b	178.5(3)
O4a	C12a	C7a	C6a	153.1(4)		C25a	C24a	C29a	C28a	-1.8(4)
O4a	C12a	C7a	C8a	-30.0(5)		C25a	C24a	C29a	C30a	177.4(4)
O4b	C11b	C4b	C3b	31.2(4)		C25a	C26a	C27a	C28a	0.3(5)
O4b	C11b	C4b	C5b	-155.3(4)		C25b	C24b	C29b	C28b	2.0(5)
N1a	C1a	C2a	C3a	-5.0(5)		C25b	C24b	C29b	C30b	-179.1(4)
N1a	C5a	C4a	C3a	-8.7(4)		C25b	C26b	C27b	C28b	1.6(6)
N1a	C5a	C4a	C11a	164.5(3)		C26a	C25a	C24a	C29a	1.0(4)
N1a	C5a	C6a	N2a	-27.0(3)		C26a	C27a	C28a	C29a	-1.1(5)
N1a	C5a	C6a	C7a	148.3(3)		C26b	C25b	C24b	C29b	-0.3(5)
N1b	C1b	C2b	C3b	5.5(5)		C26b	C27b	C28b	C29b	0.3(6)
N1b	C5b	C4b	C3b	10.6(4)		C27a	C28a	C29a	C30a	-177.2(4)
N1b	C5b	C4b	C11b	-162.9(3)		C27b	C28b	C29b	C30b	179.2(4)
N1b	C5b	C6b	N2b	25.0(4)		C28a	C29a	C30a	C31a	-0.8(6)
N1b	C5b	C6b	C7b	-151.1(3)		C28b	C29b	C30b	C31b	-2.7(5)

**N2a**	**C6a**	**C5a**	**C4a**	**152.4(3)**		**C29a**	**C30a**	**N4a**	**C34a**	**-178.8(4)**
**N2a**	**C6a**	**C7a**	**C8a**	**-7.8(5)**		**C29a**	**C30a**	**C31a**	**C32a**	**178.4(6)**
**N2a**	**C6a**	**C7a**	**C12a**	**169.1(4)**		**C29b**	**C30b**	**N4b**	**C34b**	**-177.7(3)**
**N2a**	**C10a**	**C9a**	**C8a**	**-3.3(5)**		**C29b**	**C30b**	**C31b**	**C32b**	**177.5(4)**
**N2b**	**C6b**	**C5b**	**C4b**	**-152.9(3)**		**C30a**	**N4a**	**C34a**	**C33a**	**-0.2(5)**
**N2b**	**C6b**	**C7b**	**C8b**	**7.8(5)**		**C30a**	**C31a**	**C32a**	**C33a**	**0.8(7)**
**N2b**	**C6b**	**C7b**	**C12b**	**-168.3(4)**		**C30b**	**N4b**	**C34b**	**C33b**	**-0.5(4)**
**N2b**	**C10b**	**C9b**	**C8b**	**3.3(6)**		**C30b**	**C31b**	**C32b**	**C33b**	**0.9(5)**
**N3a**	**C19a**	**C18a**	**C13a**	**-0.3(3)**		**C31a**	**C30a**	**N4a**	**C34a**	**0.3(5)**
**N3a**	**C19a**	**C18a**	**C17a**	**-179.9(3)**		**C31a**	**C32a**	**C33a**	**C34a**	**-0.6(8)**
**N3a**	**C19a**	**C20a**	**C21a**	**-0.0(4)**		**C31b**	**C30b**	**N4b**	**C34b**	**2.3(4)**
**N3a**	**C23a**	**C22a**	**C21a**	**-0.9(4)**		**C31b**	**C32b**	**C33b**	**C34b**	**0.9(5)**

Table S7. Hydrogen Atom Coordinates (Å×10^4) and Isotropic Displacement Parameters (Å^2×10^3) for Ir^{III}-p.

**Atom**	***x***	***y***	***z***	**U(eq)**
**H1wa**	**4240(30)**	**4978(3)**	**4234(15)**	**100.7(11)**
**H1wb**	**4573(14)**	**4270(20)**	**4738(3)**	**100.7(11)**
**H2wa**	**1270(40)**	**3900(20)**	**5453(8)**	**101.6(12)**
**H2wb**	**970(20)**	**4240(8)**	**4831(15)**	**101.6(12)**
**H1a**	**2415(3)**	**6074(3)**	**1776.7(17)**	**62.4(11)**
**H1b**	**6425(3)**	**8812(3)**	**2552.5(19)**	**71.4(13)**
**H2a**	**3479(3)**	**6016(3)**	**2623.0(17)**	**65.1(11)**
**H2b**	**7877(3)**	**7682(3)**	**3094.0(19)**	**71.9(13)**
**H3a**	**2551(3)**	**6042(2)**	**3507.5(16)**	**60.1(10)**
**H3b**	**7405(3)**	**6442(2)**	**3629.9(16)**	**61.2(11)**
**H8a**	**-2634(3)**	**4823(3)**	**3445.8(17)**	**64.6(11)**
**H8b**	**1465(3)**	**7287(3)**	**4144.7(19)**	**68.3(12)**
**H9a**	**-3616(3)**	**5489(3)**	**2471.7(18)**	**70.7(13)**
**H9b**	**370(3)**	**8272(3)**	**3270(2)**	**75.3(13)**
**H10a**	**-2661(3)**	**6155(3)**	**1654.5(16)**	**62.5(11)**
**H10b**	**1308(3)**	**9015(3)**	**2479.9(19)**	**69.4(12)**
**H14a**	**1522(4)**	**5751(3)**	**495.9(19)**	**69.8(12)**
**H14b**	**2457(3)**	**9582(2)**	**928.1(19)**	**69.3(12)**
**H16a**	**2997(4)**	**7623(3)**	**-338(2)**	**79.2(14)**
**H16b**	**-33(4)**	**11695(3)**	**675(3)**	**105(2)**
**H20a**	**775(3)**	**9064(3)**	**801.0(19)**	**68.4(11)**
**H20b**	**1329(8)**	**11676(5)**	**2550(4)**	**162(3)**

**H21a**	**-463(4)**	**9626(3)**	**1409(2)**	**74.5(12)**
**H21b**	**2375(11)**	**11507(7)**	**3471(5)**	**226(5)**
**H22a**	**-1617(4)**	**8844(3)**	**2050(2)**	**72.7(12)**
**H22b**	**3894(9)**	**10361(6)**	**3884(4)**	**194(4)**
**H23a**	**-1483(3)**	**7527(3)**	**2039.9(16)**	**60.9(10)**
**H23b**	**4420(6)**	**9441(4)**	**3392(3)**	**119(2)**
**H25a**	**-1908(3)**	**7999(3)**	**470.0(16)**	**61.4(10)**
**H25b**	**4702(4)**	**10809(3)**	**1971(2)**	**79.5(14)**
**H27a**	**-3346(4)**	**7208(3)**	**-705.7(19)**	**82.4(15)**
**H27b**	**6694(5)**	**10899(3)**	**516(3)**	**98.5(18)**
**H31a**	**-1160(5)**	**4553(3)**	**398(3)**	**101.1(18)**
**H31b**	**5705(3)**	**8565(3)**	**479(2)**	**75.6(13)**
**H32a**	**106(6)**	**3330(4)**	**998(3)**	**119(2)**
**H32b**	**5177(4)**	**7343(3)**	**619(2)**	**83.8(14)**
**H33a**	**1258(6)**	**3339(3)**	**1793(3)**	**112(2)**
**H33b**	**4061(4)**	**6867(3)**	**1446(2)**	**80.2(13)**
**H34a**	**1153(4)**	**4556(3)**	**1956(2)**	**82.9(14)**
**H34b**	**3494(3)**	**7623(2)**	**2073.4(19)**	**64.6(11)**

Table S8. Solvent masks information for Ir^{III}-p.

Number	X	Y	Z	Volume	Electron count	Content
1	0.518	0.133	0.367	1893.5	605.9	60 H2O

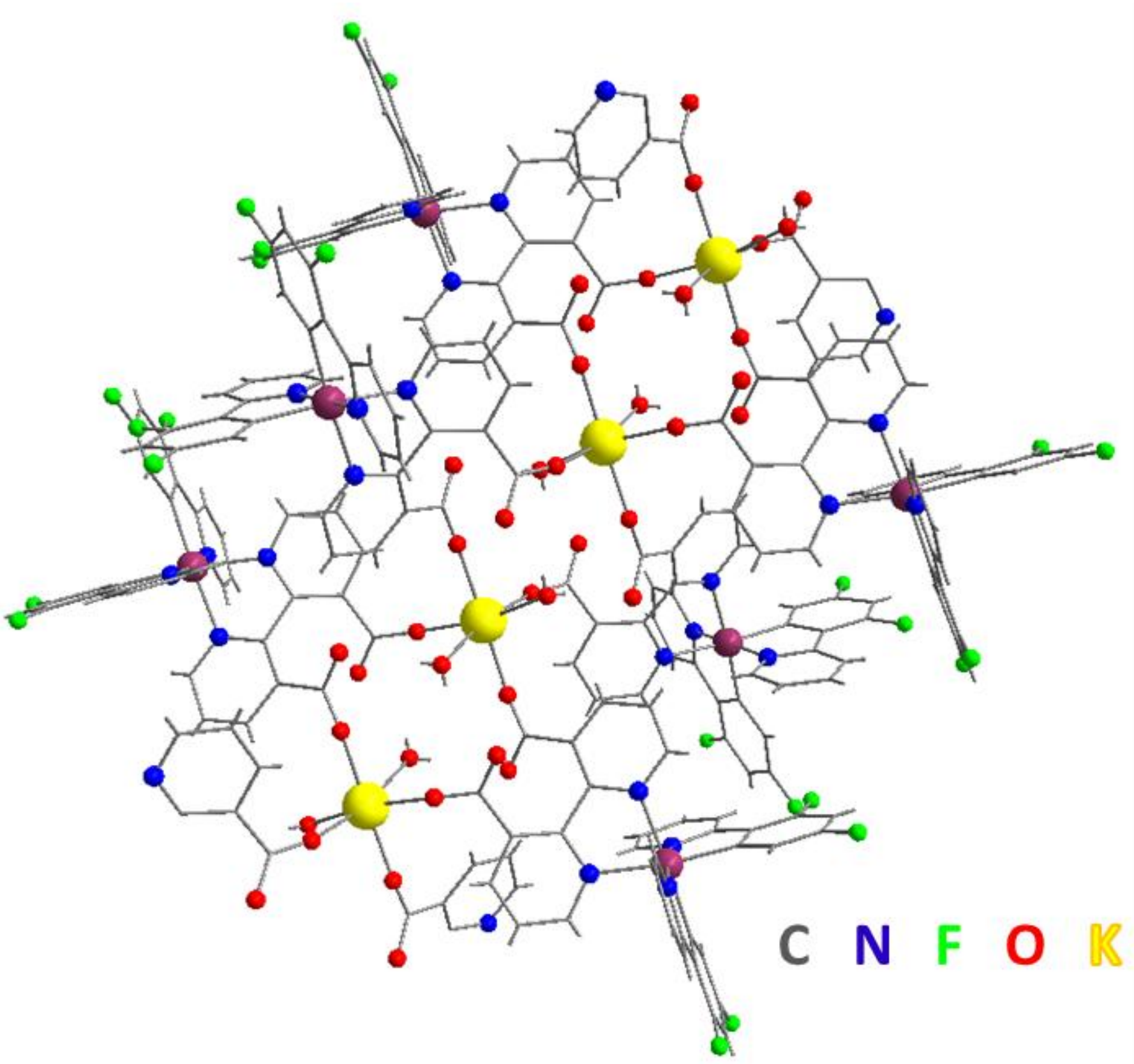

Figure S1. Crystal structure of Ir^{III}-p.

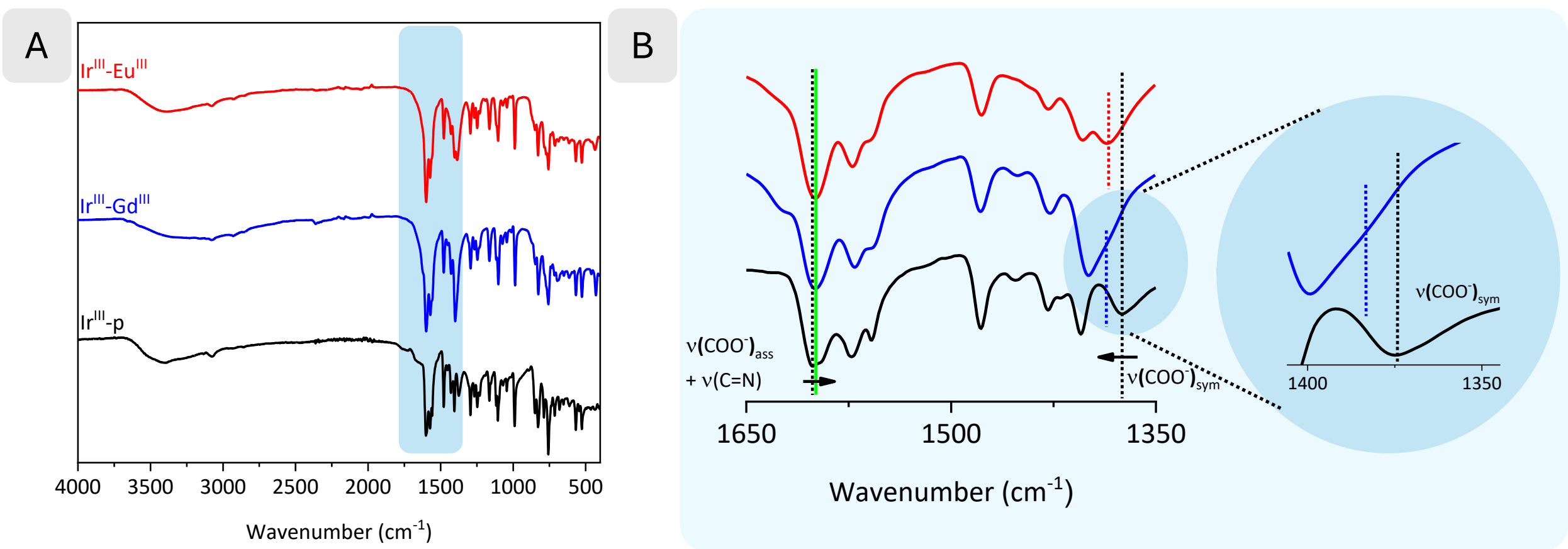

Figure S2. A) FTIR spectra of the Ir^{III}p, Ir^{III}-Gd^{III}, and Ir^{III}-Eu^{III} complexes, B) magnification of the region from 1350 cm^{-1} to 1650 cm^{-1}.

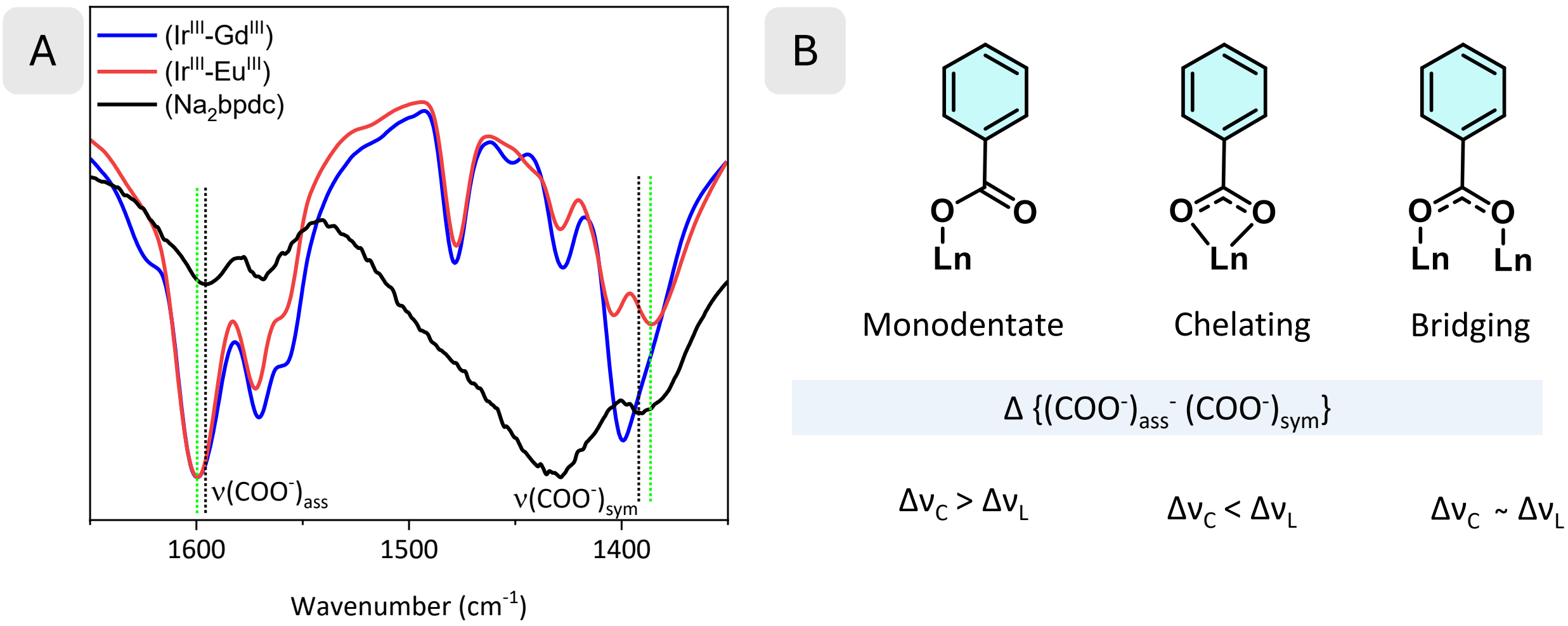

Figure S3. A) FTIR spectra of the Na_2bpdc ligand, Ir^{III}-Gd^{III}, and Ir^{III}-Eu^{III} complexes; and B) most common carboxylate coordination modes and the energy difference between vass(COO^{-}) and vsym(COO^{-}) for each coordination mode in the complexes ($\Delta\nu_c$) compared with the energy difference in the ligands ($\Delta\nu_L$).[1] To illustrate, it was used a generic carboxylic acid (benzoic acid).

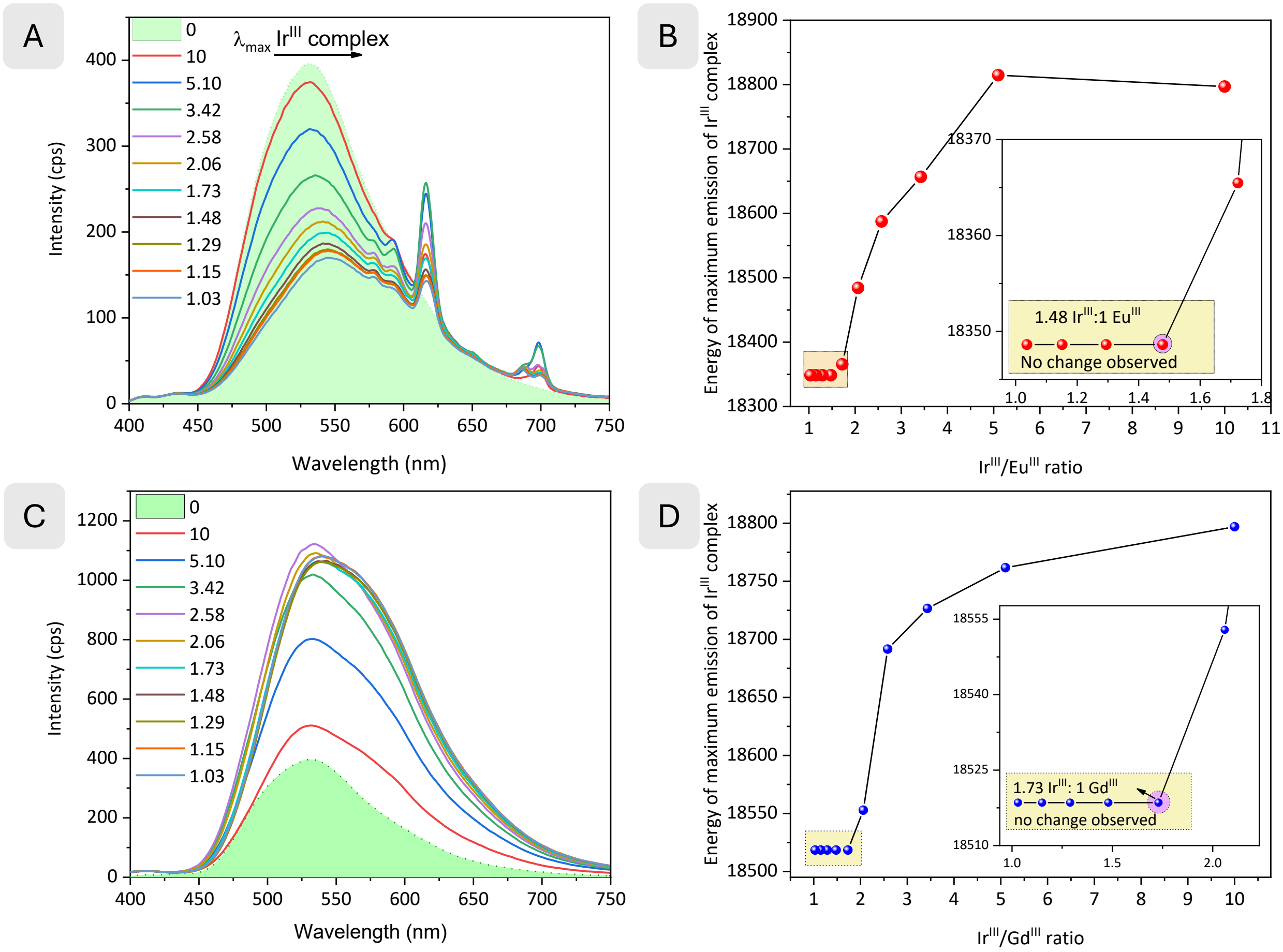

Figure S4. A) emission spectra obtained after each addition of $EuCl_3$ and B) the graph of the ratio of Ir^{III}/Eu^{III} and the energy of the maximum emission of the Ir^{III} complex. C) emission spectra obtained after each addition of $GdCl_3$ and D) the graph of the ratio of Ir^{III}/Gd^{III} and the energy of the maximum emission of the Ir^{III} complex.

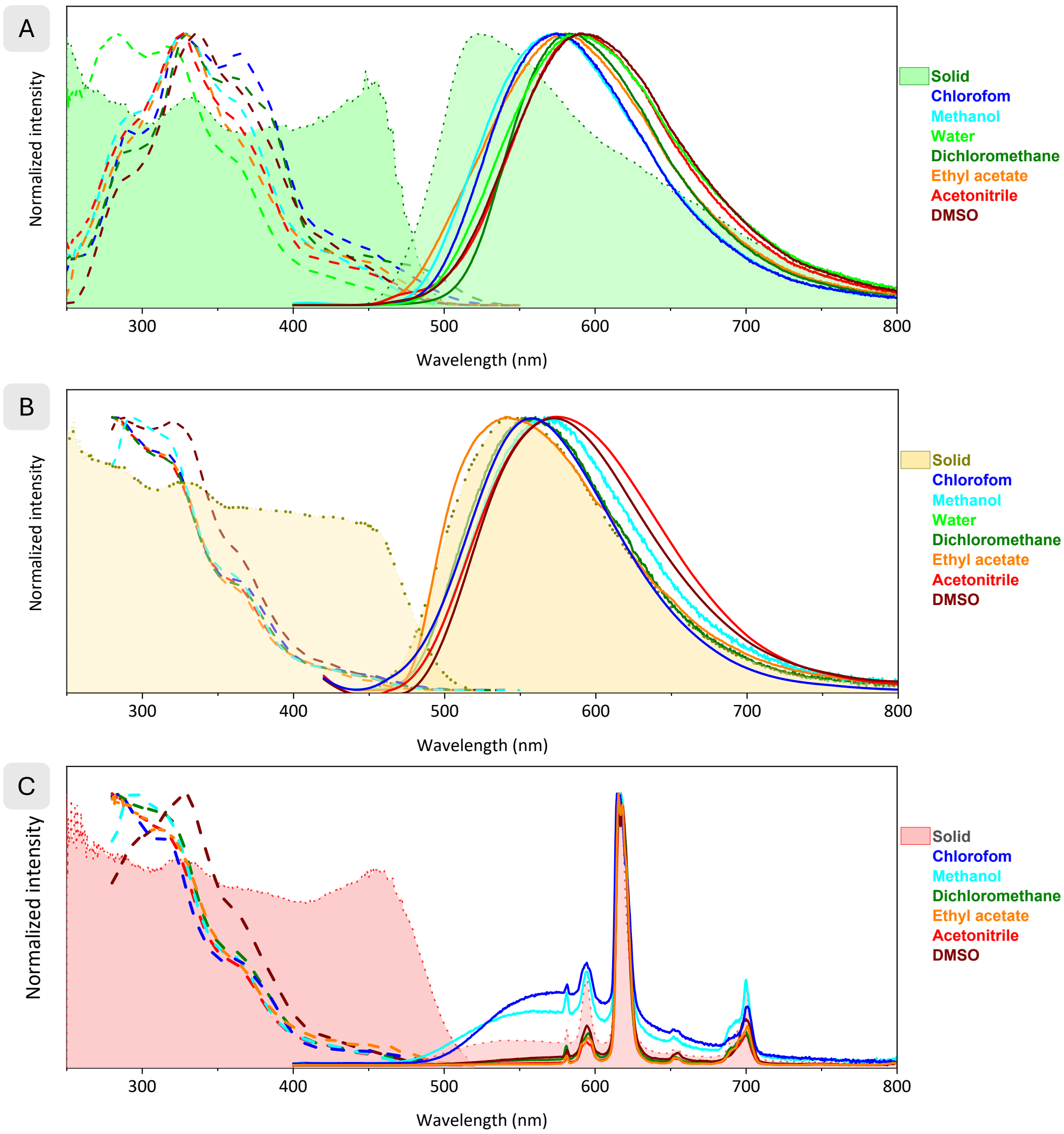

Figure S5. Excitation and emission spectra of A) Ir^{III}-p; B) Ir^{III}-Gd^{III}; and C) Ir^{III}-Eu^{III} complexes in the solid state and in various solvents. Concentration in solution of $1.0x10^{-5}$ mol·L^{-1}. All measurements were carried out with a bandpass of 2.5 nm for both Ex and Em, with an increment of 0.5 nm and an integration time of 0.5 s.

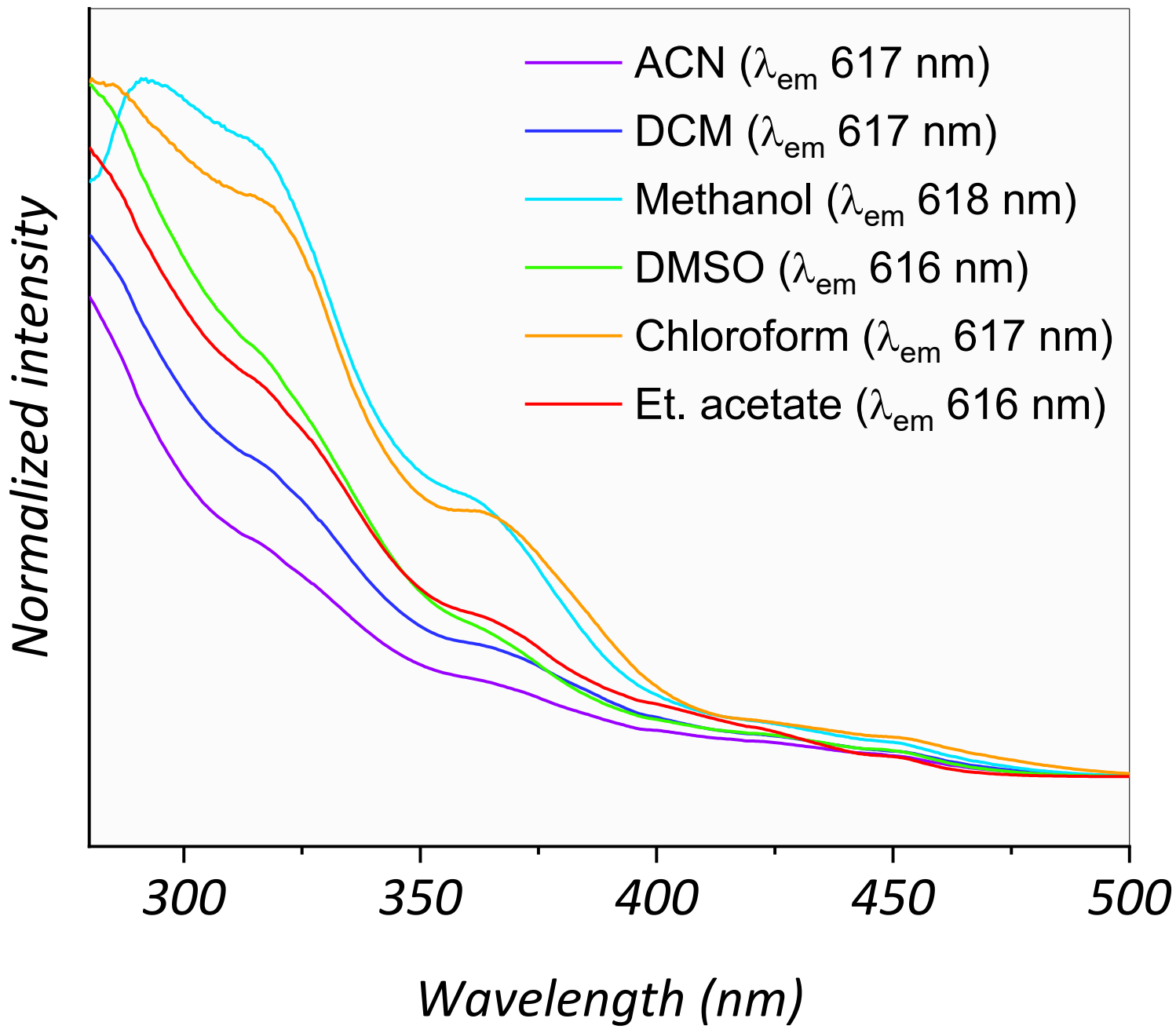

Figure S6. Excitation spectra of Ir^{III}-Eu^{III} complex in various solvents. Concentration in solution of $1.0x10^{-5}$ mol·L^{-1}. All measurements were carried out with a bandpass of 2.5 nm for both Ex and Em, with a 0.5 nm increment and an integration time of 0.5 s.

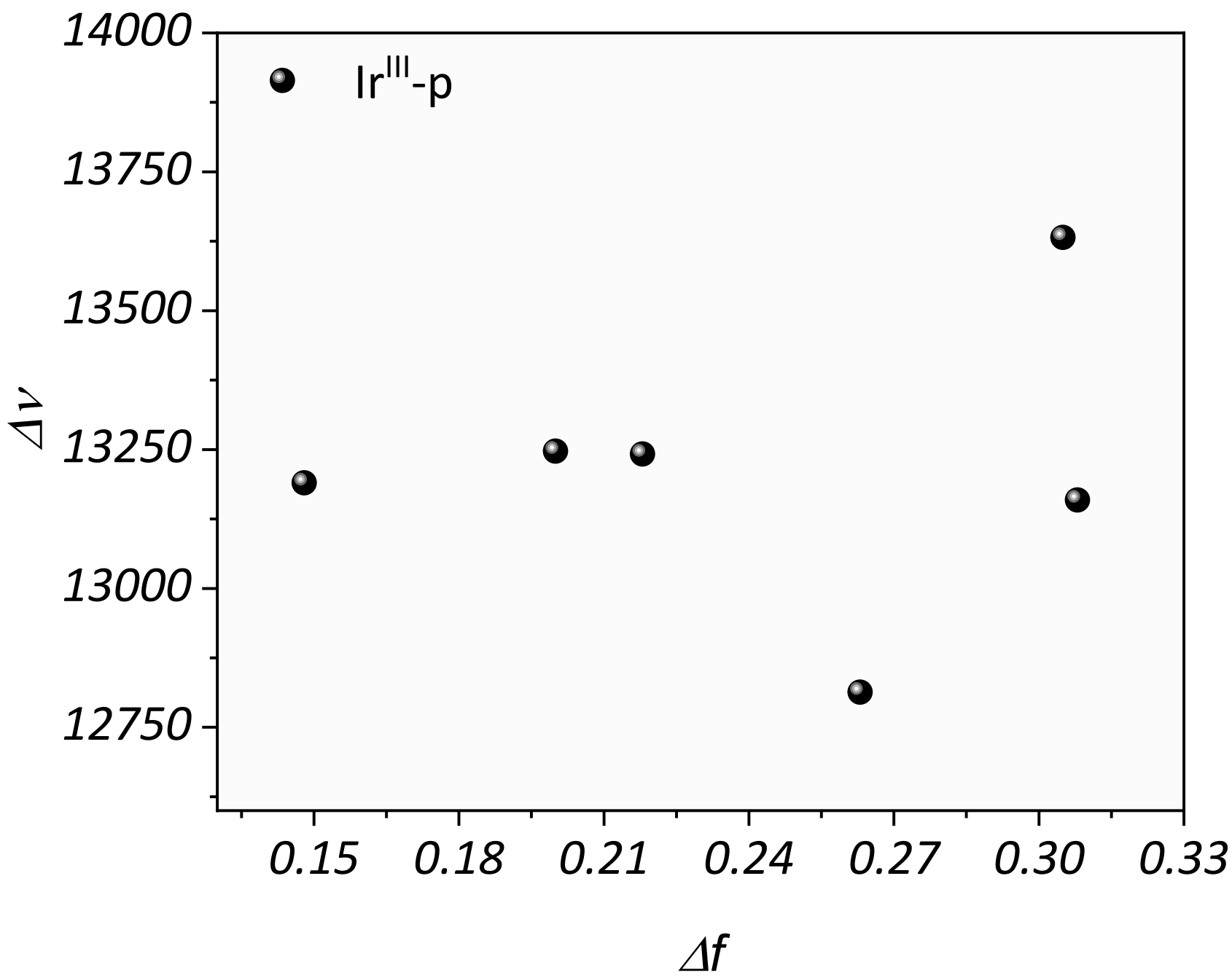

Figure S7. Lippert-Mataga plot for Ir^{III}-p bimetallic complex.

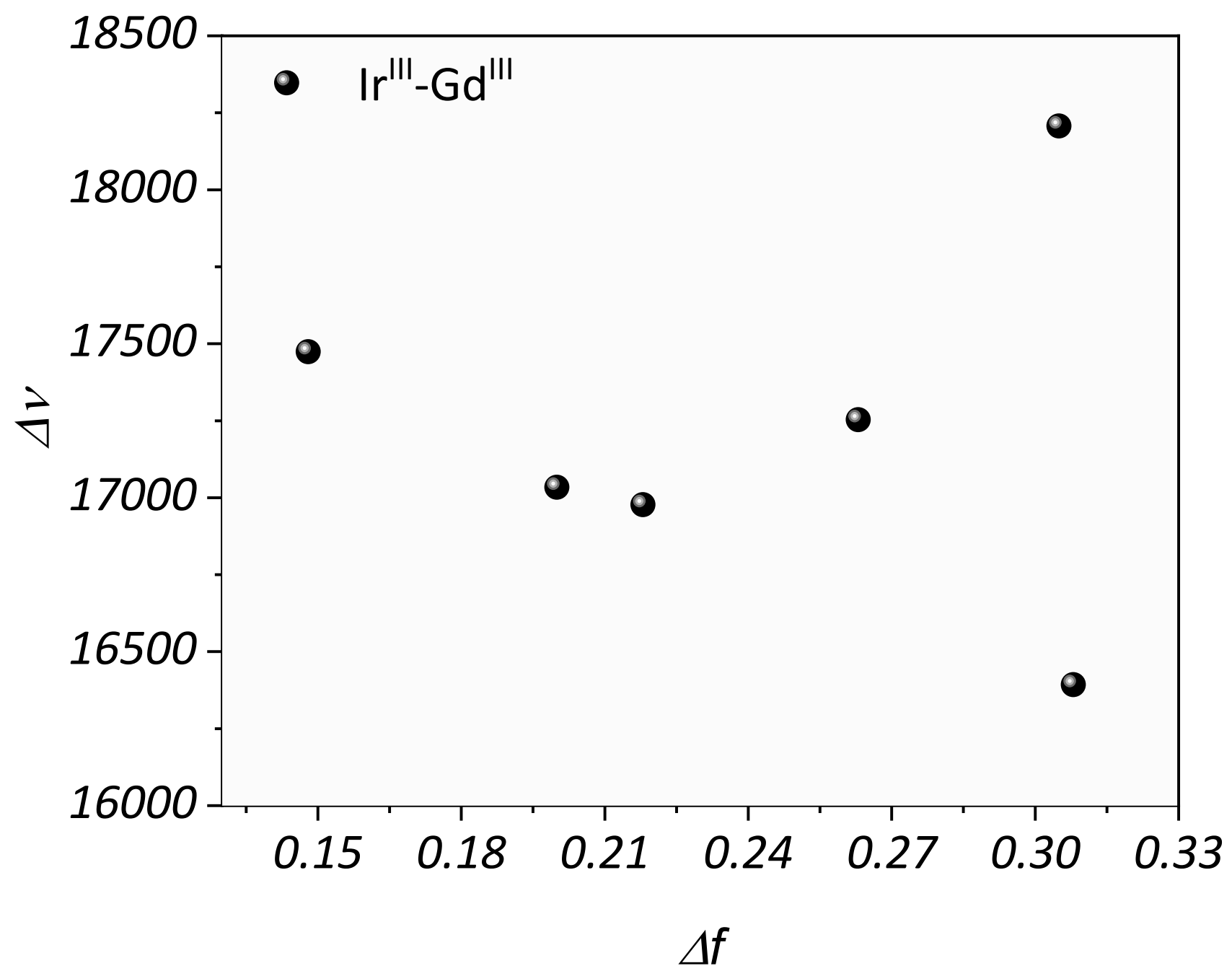

Figure S8. Lippert-Mataga plot for Ir^{III}-Gd^{III} bimetallic complex.

Table S9. Judd-Ofelt parameters (Ω_2 and Ω_4) and area ratio between emission bands related to $^5D_0 \rightarrow {}^7F_2$ and $^5D_0 \rightarrow {}^7F_1$ transitions.

	Ω_2 [10^{-20} cm^2]	Ω_4 [10^{-20} cm^2]	$R_{(0-2)/(0-1)}$
Ethyl Ac.	14.06	7.32	7.98
ACN	14.46	8.20	8.23
DMSO	9.04	8.24	5.14

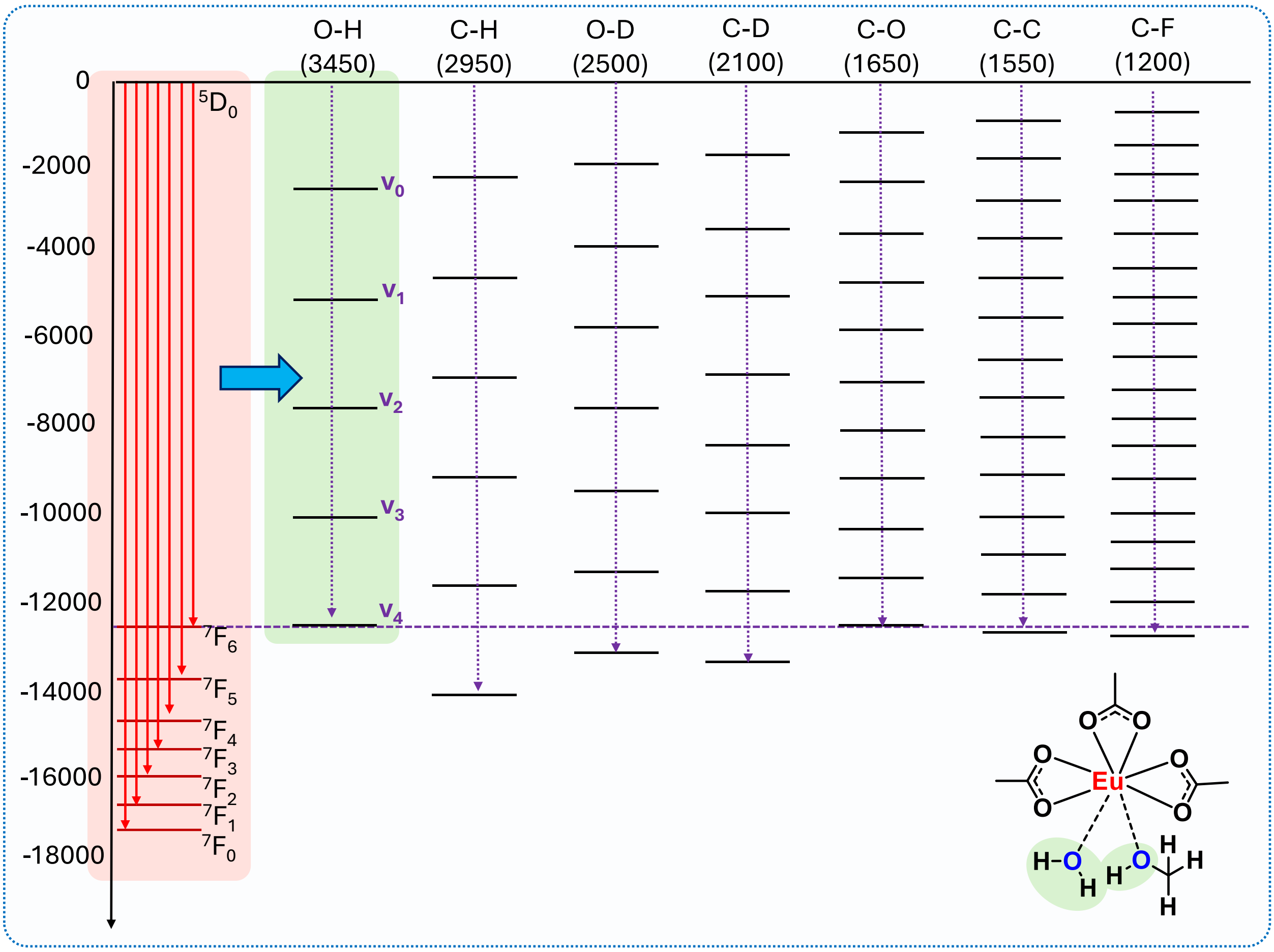

Figure S9. Schematic representation of vibrational coupling between the $^5D_0$ emitting level of Eu^{III} and selected common oscillators. The energy values are given in cm^{-1}. A generic Eu^{III} coordination sphere is included to illustrate the spatial arrangement of OH oscillators responsible for nonradiative deactivation. Adapted from [[2]].

Table S10. Judd-Ofelt parameters (Ω_2 and Ω_4) and area ratio between emission bands related to $^5D_0 \rightarrow {}^7F_2$ and $^5D_0 \rightarrow {}^7F_1$ transitions.

	Ω_2 [10^{-20} cm^2]	Ω_4 [10^{-20} cm^2]	$R_{(0-2)/(0-1)}$
Methanol	10.43	8.81	5.93
Water	-	-	-

Table S11. Judd-Ofelt parameters (Ω_2 and Ω_4) and area ratio between emission bands related to $^5D_0 \rightarrow {}^7F_2$ and $^5D_0 \rightarrow {}^7F_1$ transitions.

	Ω_2 [10^{-20} cm^2]	Ω_4 [10^{-20} cm^2]	$R_{(0-2)/(0-1)}$
DCM	11.39	6.70	6.47
Chloroform	13.33	6.50	7.58

SUPLEMENTARY INFORMATION – SECTION II

The experimental lifetime (τ) of an excited state is determined by measuring the luminescence decay as a function of time, which is related to the time required for the emissive state to decay to 1/*e* of the initial intensity. The depopulation process is described by Equation 1, where the emission intensity is proportional to the number of electrons in the excited state:

$$I = I_0 \cdot e^{-t/\tau} \textit{ (Equation 1)}$$

Depopulation can occur by radiative (A_r) or nonradiative (A_{nr}) paths. The total decay rate ($A_t = A_r + A_{nr}$) is given by the reciprocal of the lifetime, as shown in Equation 2[3]:

$$\tau^{-1} = A_r + A_{nr} \textit{ (Equation 2)}$$

The radiative decay rate is calculated by the sum of the photons emitted in each $^5D_0 \rightarrow ^7F_J$ (J = 0, 1, 2, 3, and 4) set of transitions, as shown in Equation 3 and 4:

$$A_r = A_{00} + A_{01} + A_{02} + A_{03} + A_{04} \textit{ (Equation 3)}$$

$$A_{0J} = A_{01}\left(\frac{S_{0J}}{S_{01}}\right)\left(\frac{\nu_{01}}{\nu_{0J}}\right) \textit{ (Equation 4)}$$

A_{01} is the spontaneous decay rate referring to the $^5D_0 \rightarrow ^7F_1$ transition, which is used as an internal standard because, it is allowed by the magnetic dipole and, it is not influenced by the electric field around the system. $S_{01:0J}$ and $\nu_{01:0J}$ are the area and barycenter of the $^5D_0 \rightarrow ^7F_1$ and $^5D_0 \rightarrow ^7F_J$ transitions (J = 0, 2, 3, and 4), respectively. Using the radiative and nonradiative decay rates, it is possible to determine the intrinsic emission quantum yield (Φ_{Eu}^{Eu}) of the $^5D_0$ emissive state of the complex, as shown in Equation 5[3]:

$$\Phi_{Eu}^{Eu} = \frac{A_r}{A_r + A_{nr}} \textit{ (Equation 5)}$$

[1] Socrates, G. (2001). *Infrared and Raman characteristic group frequencies: Tables and charts* (3rd ed.). John Wiley & Sons. (https://doi.org/10.1002/jrs.1238)
[2] Hasegawa, M., Ohmagari, H., Tanaka, H., & Machida, K. (2022). Luminescence of lanthanide complexes: From fundamental to prospective approaches related to water- and molecular-stimuli. Journal of Photochemistry and Photobiology C: Photochemistry Reviews, 50, 100484. (https://doi.org/10.1016/j.jphotochemrev.2022.100484)
[3] Binnemans, K. (2015). Interpretation of europium (III) spectra. *Coordination Chemistry Reviews*, *295*, 1-45. (https://doi.org/10.1016/j.ccr.2015.02.015)
